# Characterization of Two Heterogeneous Lethal Mouse-Adapted SARS-CoV-2 Variants Recapitulating Representative Aspects of Human COVID-19

**DOI:** 10.3389/fimmu.2022.821664

**Published:** 2022-02-07

**Authors:** Feihu Yan, Entao Li, Tiecheng Wang, Yuanguo Li, Jun Liu, Weiqi Wang, Tian Qin, Rina Su, Hongyan Pei, Shen Wang, Na Feng, Yongkun Zhao, Songtao Yang, Xianzhu Xia, Yuwei Gao

**Affiliations:** ^1^ Key Laboratory of Jilin Province for Zoonosis Prevention and Control, Changchun Veterinary Research Institute, Chinese Academy of Agricultural Sciences, Changchun, China; ^2^ College of Veterinary Medicine, Jilin University, Changchun, China; ^3^ School of Life Sciences, Northeast Normal University, Changchun, China; ^4^ College of Veterinary Medicine, Jilin Agricultural University, Changchun, China

**Keywords:** COVID-19, SARS-CoV-2, mouse model, mutation, pathogenesis, BLP vaccine

## Abstract

New emerging severe acute respiratory syndrome 2 (SARS-CoV-2) has caused a worldwide pandemic. Several animal models of coronavirus disease 2019 (COVID-19) have been developed and applied to antiviral research. In this study, two lethal mouse-adapted SARS-CoV-2 variants (BMA8 and C57MA14) with different virulence were generated from different hosts, which are characterized by high viral replication titers in the upper and lower respiratory tract, pulmonary pathology, cytokine storm, cellular tropism, lymphopenia, and neutrophilia. Two variants exhibit host genetics-related and age-dependent morbidity and mortality in mice, exquisitely reflecting the clinical manifestation of asymptomatic, moderate, and severe COVID-19 patients. Notably, both variants equally weaken the neutralization capacity of the serum derived from COVID-19 convalescent, but the C57MA14 variant showed a much higher virulence than the BMA8 variant *in vitro*. Q489H substitution in the receptor-binding domain (RBD) of BMA8 and C57MA14 variants results in the receptors of SARS-CoV-2 switching from human angiotensin-converting enzyme 2 (hACE2) to murine angiotensin-converting enzyme 2 (mACE2). Additionally, A22D and A36V mutation in E protein were first reported in our study, which potentially contributed to the virulence difference between the two variants. Of note, the protective efficacy of the novel bacterium-like particle (BLP) vaccine candidate was validated using the BMA8- or C57MA14-infected aged mouse model. The BMA8 variant- and C57MA14 variant-infected models provide a relatively inexpensive and accessible evaluation platform for assessing the efficacy of vaccines and novel therapeutic approaches. This will promote further research in the transmissibility and pathogenicity mechanisms of SARS-CoV-2.

## Introduction

The human coronavirus disease 2019 (COVID-19) was declared to enter a global pandemic by the World Health Organization (WHO) on March 11, 2020, as the number of infectious cases continues to increase (1). The causative agent of COVID-19, severe acute respiratory syndrome 2 (SARS-CoV-2), is a new member of the Coronaviridae family (2–6). As of January 5, 2022, there have been 290,959,019 confirmed cases of COVID-19 identified from 223 countries or territories, including 5,446,753 deaths, as reported to WHO (https://covid19.who.int). The clinical spectrum of SARS-CoV-2 infection is broad, ranging from asymptomatic, moderate, to severe pneumonia. Patients have been classified as asymptomatic carriers, individuals who are positive for SARS-CoV-2 RNA without demonstrating any clinical symptoms (7–9). Patients with moderate disease exhibit clinical signs of pneumonia (fever, cough, dyspnea, fast breathing) and have pulse oxygen saturation (SpO_2_) >90% (10). Severe cases are characterized by pneumonia and lymphopenia and at least one of the following: respiratory rate >30 breaths/min, SpO_2_ <90%, and admission to intensive care unit (ICU) for respiratory support (10–14). Severe cases, particularly the elderlies with preexisting metabolic, pulmonary, and cardiac conditions, have a higher risk of death from COVID-19 (3, 15–19).

With intensified COVID-19 pandemic, it is urgent to develop animal models to evaluate the effectiveness and safety of vaccines and therapeutic drugs before entering the clinical trial. Until very recently, several animal models of SARS-CoV-2 have been developed with varying morbidity and mortality, viral replication, and clinical diseases, including standard laboratory mice (20), human angiotensin-converting enzyme 2 (hACE2) transgenic mice (1, 21, 22), virally transduced hACE2 mice (23–25), hamsters (26, 27), ferrets (28), and non-human primates (29–32). While those reported mouse models facilitate SARS-CoV-2 infection of mice, the pathogenesis fails to model the more severe disease manifestations observed in humans accurately. Mouse adaptation and transgenesis are common strategies to develop inexpensive and convenient small animal models for human coronavirus disease, e.g., SARS-CoV (33–35), and Middle East respiratory syndrome coronavirus (MERS-CoV) (36–39). We have noted that several groups have used standard immune-competent mice and hamsters as models of COVID-19. The accessibility and the ease of use of mouse models far exceed that of ferret and non-human primate models. Golden or Syrian hamsters are susceptible to the wild-type SARS-CoV-2; however, they can recover after 1 week post-infection (40). Therefore, it is critical to develop a small animal model that manifests severe pathogenicity of acute respiratory distress syndrome (ARDS).

Clinically isolated SARS-CoV-2 is insensitive to murine angiotensin-converting enzyme 2 (mACE2) receptor and poorly replicates in mice. Several groups have developed transgenic mouse models of SARS-CoV-2 *via* different strategies to highly express hACE2 *in vivo*, including transduction using adenovirus vector (24) or adeno-associated virus vector (25), or using a variety of murine (1) or exogenous promoters (21, 22). In addition, a lethal mouse model of SARS-CoV-2 has been constructed *via* serially passaging an engineered SARS-CoV-2 derived from reverse genetics system in mice (20). Although these strategies have the advantages of accelerating the construction of SARS-CoV-2 mouse models and facilitating SARS-CoV-2 infection and replication in mice, they fail to accurately imitate the progresses of ARDS and recapitulate clinical manifestations observed in COVID-19 patients (24, 41).

To generate a small animal model reproducing the clinical characteristics of an asymptomatic infected person and moderate and severe patients of COVID-19, two mouse-adapted lethal SARS-CoV-2 variants (BMA8 and C57MA14) were obtained by serially passaging SARS-CoV-2 Wuhan01 in BALB/c and C57BL/6N mice. The two SARS-CoV-2 variants were characterized by causing dose- and age-related or host-dependent mortality and weight loss associated with high viral titers in turbinate and lung. The histopathologic findings also demonstrated pulmonary disease in mice. In particular, BMA8-infected young BALB/c or C57BL/6N mice reproduced the hallmarks of clinical asymptomatic carriers of COVID-19, while BMA8-infected aged C57BL/6N mice showed the characterization of moderate COVID-19 patients; C57MA14-infected aged or young BALB/c and C57BL/6N mice reflected the manifestation of severe COVID-19 patients. Interestingly, aged BALB/c or aged C57BL/6N mice vaccinated with Gram-positive enhancer matrix (GEM)-delivered receptor-binding domain (RBD) of SARS-CoV-2 Wuhan01 spike protein were 100% protected from BMA8 and C57MA14 variant challenge. Together, BMA8 and C57MA14 are two SARS-CoV-2 variants that can reproduce the clinical manifestations of human COVID-19 in mice, which can be used to explore the age-/sex-related and host genetics-dependent pathogenesis of SARS-CoV-2. They also provide an economical and convenient tool to assess vaccines and antiviral drugs of COVID-19.

## Results

### Adaptation of SARS-CoV-2 for an Increased Virulence in Aged BALB/c and C57BL/6N Mouse Models

To obtain mouse-adapted SARS-CoV-2 strains that are able to effectively replicate in laboratory standard mice, the human clinical isolate of SARS-CoV-2 (Beta-Cov/Wuhan/AMMS01/2020, abbreviated as Wuhan01) was serially passaged in the lungs of aged BALB/c or C57BL/6N mice as previous studies (33, 42). In each round of infection, part of the lung homogenates was collected for viral RNA detection and infectious titer test. The viral RNA copies and TCID_50_ in the lung gradually increased to a peak in the fourth passage (P4) and remained at a similar level from P5 to P9 in aged BALB/c mice. The peak observed in P9 was maintained at the similar level from P8 to P15 in aged C57BL/6N mice (**Figures S1A, B, F, G**). To screen for the virulence of different generations of virus in mice, three groups of aged BALB/c mice (n = 8) were infected independently with lung homogenates harvested from infected BALB/c mice of P1, P4, and P8, and four groups of aged C57BL/6N mice (n = 8) were infected independently with lung homogenates harvested from infected C57BL/6N mice of P2, P6, P10, and P14. Weight change, body temperature, and mortality were observed daily. In aged BALB/c mice, deaths were not observed in P1/P4-inoculated groups (**Figure S1C**), but weight loss and persistent hypothermia were observed in P4-inoculated mice from 1 day post infection (dpi) (**Figures S1D, E**). Fatality was noted in P8-inoculated group at 3 dpi, and all mice succumbed to P8 adapted virus with significant weight loss and decreased temperature at 4 dpi (**Figure S1C**). Nevertheless, in aged C57BL/6N mice, deaths were not observed following inoculation with P2 or P6 (**Figure S1H**), only slight weight loss was observed in P6-inoculated mice between 1~7 dpi (**Figure S1I**), and the body temperature did not show any abnormality in P2/P6-inoculated mice (**Figure S1E**). However, increased morbidities and mortalities were noted in P10-inoculated mice at 6 dpi and in P14-inoculated mice at 4 dpi, with 62.5% (5/8) lethality in P10-inoculated mice at 8 dpi and 100% lethality in P14-inoculated mice at 6 dpi (**Figure S1H**). Overall, the infectivity and pathogenicity of the SARS-CoV-2 clinical isolate were enhanced after serial passaging in two outbreed mouse strains.

### Characterization of the Mouse-Adapted SARS-CoV-2 BMA8 and C57MA14

**Figure 1:**
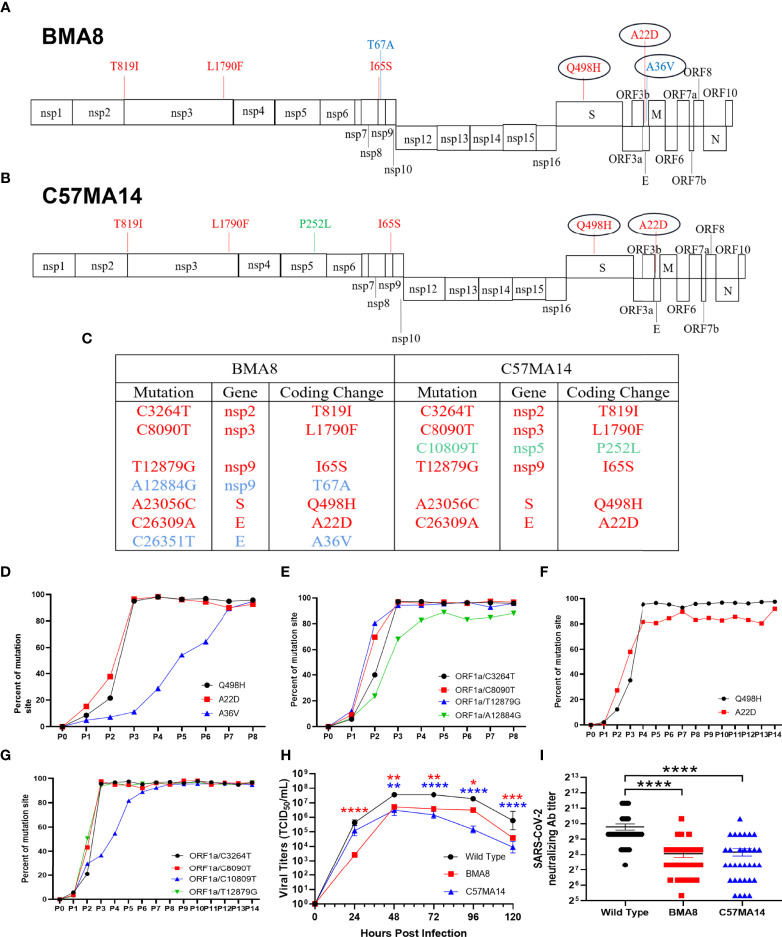
Two mouse-adapted strains of severe acute respiratory syndrome 2 (SARS-CoV-2) from outbred mice carry unique amino acid substitution. Schematic diagram of SARS-CoV-2 genome and all the adaptive mutations of amino acid identified in BMA8 **(A)** and C57MA14 **(B)** compared with the SARS-CoV-2 Wuhan01. “nsp”, nonstructural protein; “S”, Spike Protein; “ORF”, Open Reading Frame; “E”, Envelope Protein; “M”, Membrane Protein; “N”, Nucleocapsid Protein. **(C)** Table of mutations present in plaque purified mouse-adapted SARS-CoV-2 BMA8 and C57MA14. Red font showed the same mutations in both mouse-adapted variants. Blue font showed the mutations only occurring in SARS-CoV-2 BMA8. Green font showed the mutation only occurring in SARS-CoV-2 C57MA14. The proportions of all the amino acid mutations in both mouse-adapted strains were calculated in each passage **(D–G)**. One-step growth curves of SARS-CoV-2 Wuhan01, BMA8, and C57MA14 were measured in Vero E6 cells. n = 3 technical replicates for each group, representative of 2 independent experiments **(H)**. Neutralization capabilities of the serum samples from 32 SARS-CoV-2-infected individuals were measured with BMA8, C57MA14, and SARS-CoV-2 Wuhan01 **(I)**. (*P < 0.05, **P < 0.01, ***P < 0.001, ****P < 0.0001).

To identify the mutations in the BMA8 and C57MA14 that are associated with the lethal phenotype, BMA8 and C57MA14 were cloned by performing two rounds of terminal dilution in Vero E6 cells. Deep sequencing of mouse-adapted SARS-CoV-2 from each passage was performed and compared with the parental sequence of SARS-CoV-2 Wuhan01. Seven nucleotide substitutions were identified in aged BALB/c mouse-adapted SARS-CoV-2, and six nucleotide substitutions emerged in aged C57BL/6N mouse-adapted SARS-CoV-2. All the mutations are distributed in the Open Reading Frame (ORF) 1a, S, and E gene (**Figures 1A, B**). Subsequently, deep sequencing of purified SARS-CoV-2 P8 clone (BMA8) from the aged BALB/c mice and SARS-CoV-2 P14 clone (C57MA14) from the aged C57BL/6N mice was performed. Five mutations, including T819I (nsp2), L1790F (nsp3), I65S (nsp9), Q498H (S), and A22D (E), emerged in both strains. In addition, the BMA8 variant had two more mutations at T67A (nsp9) and A36V (E), and C57MA14 variant had one more mutation at P252L (nsp5) (**Figure 1C**). To track the adapted evolution of BMA8 and C57MA14 variants, the order of the mutations was further analyzed by deep sequencing. As expected, A23056C, C26309A, and C26351T mutations emerged in P1, and the proportion of Q498H (S), A22D (E), and A36V (E) mutations and other four mutations (ORF 1a) in aged BALB/c mouse-adapted virus gradually increased following the subsequent passages (**Figures 1D, E**), so did the Q498H (S), A22D (E), and other four mutations (ORF 1a) in aged C57BL/6N mouse-adapted virus (**Figures 1F, G**). Hence, all the above mutations were predicted to cause the enhanced infectivity and pathogenicity of BMA8 and C57MA14 in mice. Furthermore, the infectivity of BMA8 and C57MA14 in the Vero E6 cells was attenuated in comparison to the SARS-CoV-2 clinical isolate (**Figure 1H**), suggesting a reduced fitness in non-human primate cells. In comparison to the parental SARS-CoV-2, these two variants weakened the neutralizing capability of COVID-19 convalescent serum by 4- to 8-fold (**Figure 1I**), suggesting that the mutations in BMA8 and C57MA14 contributed to the viral escape from neutralization.

### SARS-CoV-2 BMA8 and C57MA14 Cause Severe COVID-19 Disease in Aged Mice

To investigate the dose-dependent pathogenicity of BMA8 and C57MA14, several groups of 9-month-old female BALB/c or C57BL/6N mice (n = 8) were infected with 10, 10^2^, 10^3^, 10^4^, and 10^5^ TCID_50_ of BMA8 or C57MA14. The mortality and the weight loss were observed daily. Dose-dependent increase in mortality and the weight loss over 9 days with BMA8 or C57MA14 were observed (**Figures S2A, B**). All aged BALB/c mice infected with 10^4^ or 10^5^ TCID_50_ of BMA8 died or lost over 30% of their initial body weight at 6 dpi (**Figure S2A**), and the 50% lethal dose (LD_50_) was 10^3^ TCID_50_. Similarly, all aged C57BL/6N mice infected with 10^5^ TCID_50_ SARS-CoV-2 C57MA14 viruses succumbed to infection or lost over 30% of their initial body weight by 5~7 dpi (**Figure S2B**), and the LD_50_ was 10^4^ TCID_50_.

**Figure 2:**
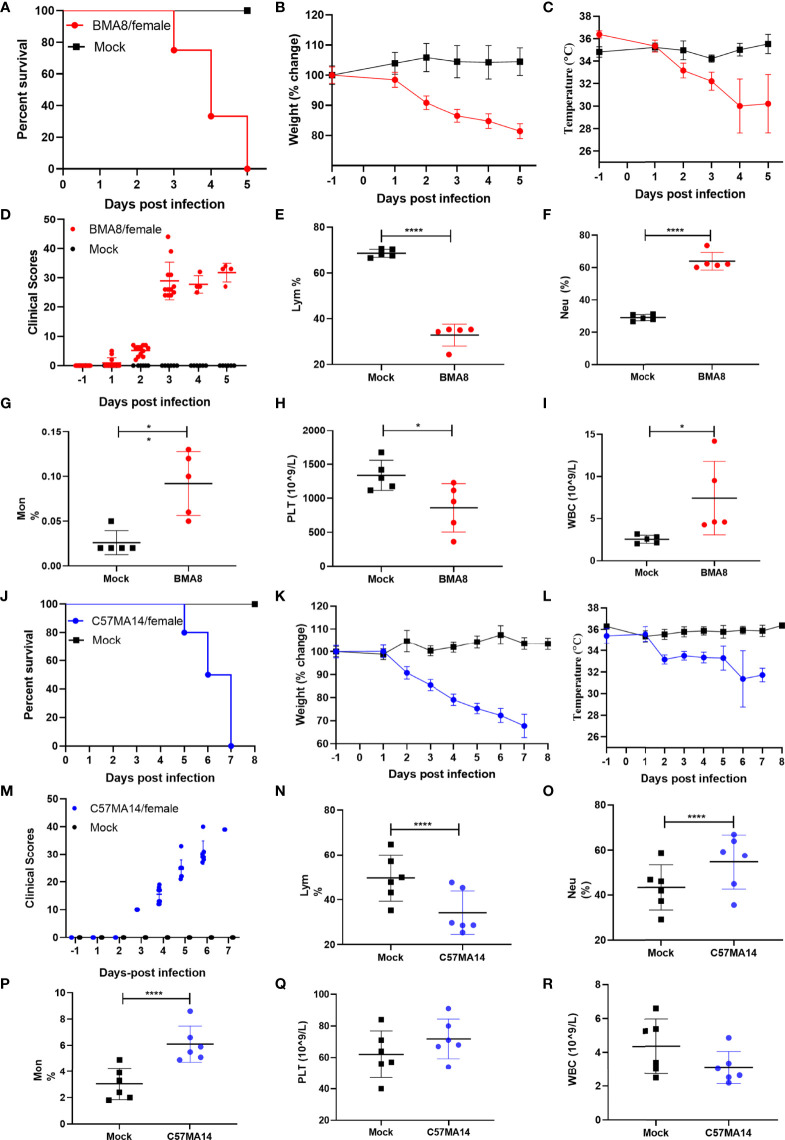
Mouse-adapted strains severe acute respiratory syndrome 2 (SARS-CoV-2) BMA8 and C57MA14 cause severe disease in aged BALB/c and C57BL/6N mice. Nine-month-old female BALB/c mice or C57BL/6N mice were mock infected (n = 13) or infected with 50 LD_50_ SARS-CoV-2 BMA8 or C57MA14 (n = 13), respectively. The survival rate, weight change, body temperature, and clinical scores of BALB/c mice were monitored daily after SARS-CoV-2 BMA8 infection **(A–D)** (n = 8) or those parameters of C57BL/6N mice were monitored daily after SARS-CoV-2 C57MA14 infection **(J–M)** (n = 8). The hematological values of BALB/c mice were analyzed, including lymphocyte percentage (LYM%), neutrophil percentage (Neu%), monocyte percentage (Mon%), platelet (PLT) count, and white blood cell (WBC) count, at 3 dpi after SARS-CoV-2 BMA8 infection **(E–I)** (n = 5), or those measurement indicators of C57BL/6N mice were also observed daily after SARS-CoV-2 C57MA14 (N-R) (n = 5). Data are presented as mean ± SEM. (*P < 0.05, ****P < 0.0001).

**Figure 3:**
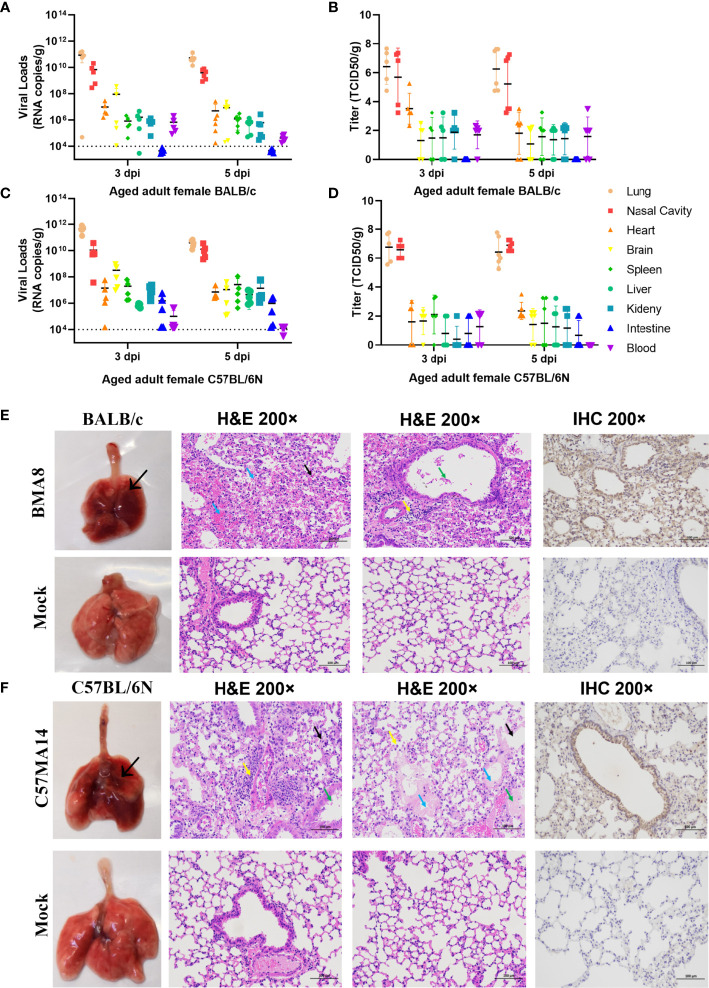
Tissue distribution of the viral RNAs and the pathological lung lesions in passaged mice infected with the mouse-adapted severe acute respiratory syndrome 2 (SARS-CoV-2) BMA8 and C57MA14. To screen virus replication, groups of aged BALB/c mice (n = 10) or aged C57BL/6N mice (n = 10) were infected with 50 LD_50_ of SARS-CoV-2 BMA8 or C57MA14, and infected mice were sacrificed at 3 dpi and 5 dpi (n = 4 or 5), respectively. The blood and internal organs were harvested to analysis the viral RNA loads by qRT-PCR and TCID_50_ **(A–D)**. Data are presented as mean ± SEM (n = 5). The gross pathology and histopathology of lungs from 9-month-old female BALB/c mice intranasally inoculated with 50 LD_50_ of SARS-CoV-2 BMA8 or PBS (Mock) at 3 dpi **(E)**. The thickened and narrowed alveolar cavity, the congested capillaries in the alveolar wall (black arrow), the infiltration of neutrophils (yellow arrow), and perivascular edema and a small amount of inflammatory cell infiltration (yellow arrow) were observed in lung tissues at 3 dpi after viral infection in BALB/c mice **(E)**. Lung tissue changes of infected C57BL/6N mice are characterized by exudation in airway (green arrow), thickened alveolar wall (black arrow), alveolar space stenosis, macrophage infiltration (yellow arrow), inflammatory cell infiltration (yellow arrow), and perivascular edema and exudation of protein-like substance in alveolar sac (blue arrow). Similarly, the gross pathology and histopathology of lungs from 9-month-old female C57BL/6N mice intranasally inoculated with 50 LD_50_ of SARS-CoV-2 C57MA14 or PBS (Mock) at 3 dpi **(F)**. Photographs of gross pathological lungs shown in left panels. Local lesions are indicated by arrows. Hematoxylin and eosin stain (H&E) shown in middle panels. Right panels show immunohistochemistry (IHC) labeling against SARS-CoV-2. Scale bar, 100 µm.

To investigate the pathogenicity of BMA8 or C57MA14 in aged BALB/c or C57BL/6N mice, all animals were infected with 50 LD_50_ BMA8 or C57MA14 and monitored daily for signs of disease. The deaths were noted in aged BALB/c mice after infection with BMA8 at 3 dpi, and 100% of inoculated mice died at 5 dpi (**Figure 2A**). The time to death was postponed to 5 dpi and 100% of inoculated mice died at 7 dpi in aged C57BL/6N mice, which was slightly longer than that in aged BALB/c mice infected with BMA8 (**Figure 2G**). Next, changes in blood counts, the growth kinetics, and tissue tropism of BMA8 or C57MA14 in aged BALB/c or C57BL/6N mice were also examined. The BMA8-infected BALB/c mice and C57MA14-infected C57BL/6N mice showed typical severe characteristics of COVID-19 infection, with significant decreases in the percentage of lymphocytes (LYM%) (**Figures 2E, N**) and a marked increase in the percentage of neutrophils (Neu%) (**Figures 2F, O**) and monocytes (Mon%) (**Figures 2G, P**). There are some differences in platelet (PLT) count and white blood cell (WBC) count in infected mice. The BMA8-infected BALB/c mice showed a decrease in PLT count and an increase in WBC count, but C57MA14-infected C57BL/6N mice showed a slight increase in PLT count and a moderate decrease in WBC count. The replication kinetics and tissue tropism of BMA8 virus or C57MA14 virus in aged BALB/c or C57BL/6N mice were then examined at 3 and 5 dpi (**Figures 3A, B**). There was a high level of viral RNA load in the lung and turbinate at 3 and 5 dpi in both BALB/c and C57BL/6N mice, with peak viral RNA loads of ~10^11^ copies/g at 3 dpi. This value is higher than that from other mouse-adapted SARS-CoV-2 with ~10^10^ copies/g (42, 43). In addition to the respiratory organs, total RNA was also detected from the heart, brain, spleen, liver, kidney, intestine, and blood of infected aged BALB/c mice and C57BL/6N mice. The viral RNAs were detected in the heart, brain, spleen, liver, and kidney of infected mice, and the live viruses were also identified in above internal organs. Obviously, high viral RNA copies were confirmed in lungs (10^10^~10^12^ copies/g) and turbinates (10^7^~10^11^ copies/g) (**Figures 3A, C**). The viral RNA was detected in blood but not in the intestines of BMA8-infected aged BALB/c mice, the TCID_50_ results were in accordance with the viral RNA test (**Figures 3A, B**). However, the viral RNAs and live viruses were not detectable in blood and could be detected in the intestine in C57MA14-infected aged C57BL/6N mice (**Figures 3C, D**).

**Figure 4:**
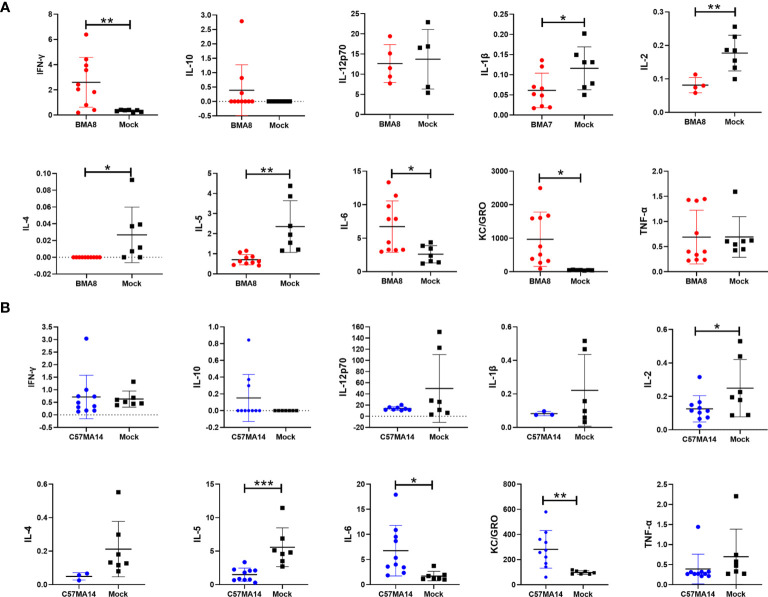
Cytokine and chemokine production in serum of aged mice infected with the mouse-adapted severe acute respiratory syndrome 2 (SARS-CoV-2) BMA8 or C57MA14. Groups of aged BALB/c mice (n = 10) **(A)** or aged C57BL/6N mice (n = 10) **(B)** were infected with 50 LD_50_ SARS-CoV-2 BMA8 or C57MA14, and the sera were harvested at 3 dpi to detect the cytokine and chemokine profiles in serum, respectively. Groups of aged BALB/c mice (n = 10) **(A)** and aged C57BL/6N mice (n = 10) **(B)** were intranasally inoculated with the same volume of PBS as control. Data are presented as mean ± SEM. (*P < 0.05, **P < 0.01, ***P < 0.001).

Subsequently, the gross pathology and histological assays were evaluated to check the pathological changes in lungs at 3 dpi (**Figures 3E, F**). Both aged BALB/c mice and aged C57BL/6N mice had macroscopically detectable discoloration, characterized by gross pulmonary edema, focal hemorrhage, consolidation, and lung bullae after BMA8 or C57MA14 infection, while uninfected mice had no visible changes. Both infected BALB/c and C57BL/6N mice presented with severe pneumonia, characterized by thickened alveolar septa, alveolar damage, and inflammatory cell infiltration. Additionally, a thick exudation of protein-like substance was found in the alveolar sac (**Figures 3E, F**). Besides lung tissues, other major organs, including the heart, brain, spleen, kidney, and intestine, were also collected to investigate the pathology at 3 dpi. These organs from infected aged BALB/c or C57BL/6N mice presented different degrees of tissue damage (**Figures S4, S5**), such as germinal center, nuclear pyknosis or fragmentation, extramedullary hematopoiesis foci in the red pulp, and brownish yellow granules in the red pulp in spleen (**Figure S4C**). The observation is consistent with viral RNA loads in organs. Meanwhile, SARS-CoV-2 nucleocapsid was detected in the lung tissue by immunohistochemistry (IHC) staining, especially in conducting airway epithelia and in the alveoli, consistent with mouse ACE2 distribution patterns in lungs (44). Notably, inflammatory cytokines in mouse sera, including Interleukin 6 (IL-6) and Keratinocyte chemoattractant (KC)/growth-regulated oncogene (GRO), were remarkably upregulated, while IL-2 and IL-5 were significantly downregulated after BMA8 or C57MA14 infection in mice compared to that of the uninfected group (**Figures 4A, B**), as seen in the severe COVID-19 patient serum. Besides those, the level of interferon γ (IFN-γ), IL-1β, and IL-4 was higher in BMA8-infected BALB/c mice than that in uninfected mice, while these cytokines showed no significant difference between C57MA14-infected mice and healthy control (**Figures 4A, B**), and this may be one of the un-neglected factors to contribute to the earlier death in BMA8-infected BALB/c mice.

For characterizing the sex-associated pathogenesis of BMA8 and C57MA14 in aged BALB/c and C57BL/6N mice, 9-month-old male BALB/c mice were infected with 50 LD_50_ BMA8 and 9-month-old male C57BL/6N mice were infected with 50 LD_50_ C57MA14. The deaths were noted in both mice at 4 dpi, and 100% of inoculated mice died at 7 or 8 dpi (**Figures S3A, D**). Weight loss was observed by 2 dpi (**Figures S3B, E**), and viral RNA loads peaked at ~10^11^ copies/g in lungs and ~10^9^ copies/g in turbinates of BALB/c mice at 3 dpi, while ~10^11^ copies/g in lungs and ~10^8^ copies/g in turbinates of C57BL/6N mice (**Figures S3C, F**). The above results were similar to those of aged female BALB/c or C57BL/6N mice (**Figures 2**, **3**). These results demonstrated no significant gender-based susceptibility difference between the female and the male mice.

### Evaluating the Age-Related and Host Genetic-Dependent Pathogenicity of BMA8 and C57MA14

**Figure 5:**
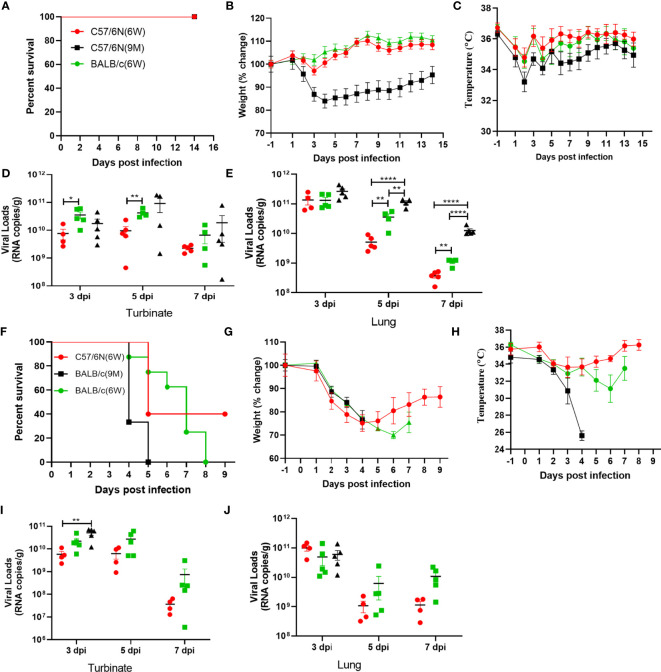
Characterization of age-related and host genetics-dependent mortality and morbidity of severe acute respiratory syndrome 2 (SARS-CoV-2) BMA8 and C57MA14 in mice. To characterize the pathogenesis of SARS-CoV-2 BMA8 associated with age and host genetics, groups of 6-week-old female C57BL/6N mice, 6-week-old female BALB/c mice, and 9-month-old female C57BL/6N mice were infected intranasally with 50 LD_50_ of SARS-CoV-2 BMA8 in a volume of 50 µl. The survival rate, weight change, and body temperature in each group (n = 8) were monitored daily after infection **(A–C)**. At 3, 5, and 7 dpi, five mice of each group were euthanized, and turbinates and lungs were sampled for virus RNA loads by qRT-PCR **(D, E)**. Similarly, to assess the pathogenesis of SARS-CoV-2 C57MA14, groups of 23 6-week-old female C57BL/6N mice, 6-week-old female BALB/c mice, and 9-month-old female BALB/c mice were infected intranasally with 50 LD_50_ of SARS-CoV-2 C57MA14 in a volume of 50 µl. The survival rate, weight change, body temperature, and clinical score in each group (n = 8) were monitored daily after infection **(F–H)**. At 3, 5, and 7 dpi, five mice of each group were euthanized, and turbinates and lungs were sampled for viral RNA load test by qRT-PCR **(I, J)**. Data are presented as mean ± SEM. (*P < 0.05, **P < 0.01,****P < 0.0001).

Next, we investigated the age-related and host genetic-dependent pathogenic potential of BMA8 and C57MA14. Here, 10-week-old female BALB/c or C57BL/6N mice and 9-month-old female C57BL/6N mice were infected with 50 LD_50_ BMA8, while 10-week-old female BALB/c or C57BL/6N mice and 9-month-old female BALB/c were infected with 50 LD_50_ C57MA14. The mortality, weight loss, body temperature, and viral loads in turbinates and lungs were evaluated. In BMA8-infected groups, all mice survived (**Figure 5A**), but aged C57BL/6N mice showed a significant weight loss by 2~4 dpi and a subsequent weight gain by 5~14 dpi without significant temperature change (**Figures 5B, C**). The results of viral RNA test suggested that BMA8 virus was cleared with the faster speed in 6-week-old C57BL/6N mice than that in the 6-week-old BALB/c mice, but the kinetics of infectious virus clearance was slower in 9-month-old C57BL/6N mice (**Figures 5D, E**). After inoculating with 50 LD50 C57MA14, 100% of young BALB/c mice died by 8 dpi and 100% of aged BALB/c mice died by 4~5 dpi, whose time of death is advanced in comparison to that of aged C57BL/6N mice (4~7 dpi) and young BALB/c mice (4~8 dpi). Here, 60% of young C57BL/6N mice succumbed to C57MA14 infection (**Figure 5F**). The clinical findings also showed a significant weight loss (**Figure 5G**) and a decreased temperature (**Figure 5H**) in all mouse groups. The viral RNA test indicated that the virus was also cleared with a faster speed in 6-week-old C57BL/6N mice than that in BALB/c mice, but the viral load was kept higher in C57MA14-infected BALB/c mice no matter which age they are (**Figures 5I, J**). To further study the influence of different genes during infection with SARS-CoV-2, we also tested the two variants in C57Bl/6J mice, in which most of the KO transgenic mice have this background. We found that the pathogenicity of BMA8 and C57MA14 in C57BL/6J mice was very similar to that of C57BL/6N mice. For the survival rate (**Figure S6A**), weight change (**Figure S6B**), and viral load (**Figures S6C, D**), there is no significant difference between C57BL/6N and C57BL/6J mouse models no matter for BMA8 virus or C57MA14 virus, although the sequence differences between C57BL/6N and C57BL/6J included 34 coding single-nucleotide polymorphisms (SNPs), 2 coding small indels (insertion or deletion mutations), 146 noncoding SNPs, and 54 noncoding small indels. This may be due to the fact that both types of mice are derived from C57BL/6 and have highly similar genetic backgrounds. The susceptibility of the two subtypes of C57BL/6 mice to SARS-CoV-2 and the mechanism of immune response to the virus should be very similar. Together, these results illustrated that BMA8 and C57MA14 cause age-related and host genetic-dependent diseases with various motility and morbidity in mice, and the pathogenicity of C57MA14 was much stronger than that of BMA8 in the same mouse strain.

### Increased Infectivity and Pathogenicity Associated With New Receptor mACE2

**Figure 6:**
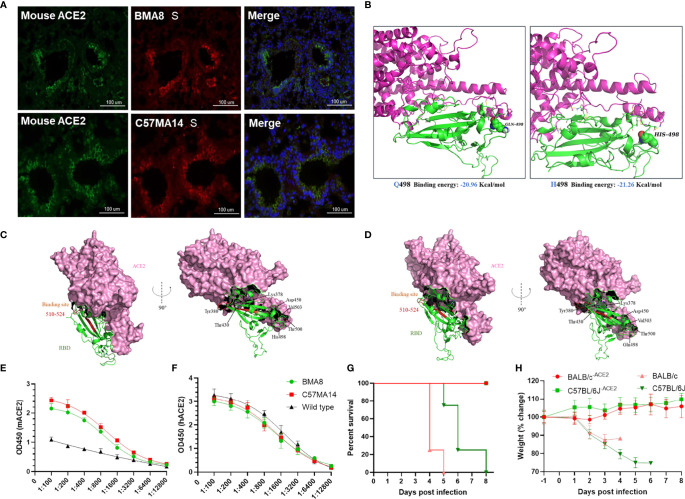
Q498H substitution increased the binding affinity between receptor-binding domain (RBD) and murine angiotensin-converting enzyme 2 (mACE2). **(A)** Multiplex immunofluorescence staining of mouse lung section, severe acute respiratory syndrome 2 (SARS-CoV-2) (red), mACE2 (green), nuclei (blue). **(B)** Homology modeling of mouse ACE2 (purple) in complex with SARS-CoV-2 variant RBD (green) with Q498 (left) or H498 (right). **(C, D)** Contribution of each amino acid to the binding free energy in complex of RBD containing Q498 or H498 binding with mACE2. **(E, F)** ELISA for detecting the binding ability between SARS-CoV-2 RBD and ACE2. **(G, H)** Survival rate and weight change of BALB/c^-ACE2^ mice (n = 3) and C57BL/6J^-ACE2^ mice (n = 3), while normal BALB/c mice (n = 4) and C57BL/6J mice (n = 4) were as control.

To investigate potential mutations in SARS-CoV-2 BMA8 and C57MA14 associated with increased infectivity, immuno-fluorescence staining supported colocalization of mouse ACE2 and SARS-CoV-2 S protein in the lungs of SARS-CoV-2-infected mice (**Figure 6A**). The A23056C mutation resulted in a Q498H amino acid substitution in the RBD of the S protein; structural remodeling suggested that the Q498H substitution in the RBD of SARS-CoV-2 S protein increased the binding affinity of the protein to mACE2 (**Figure 6B**). Protein structure prediction analysis indicated that contribution of each amino acid to the binding free energy in complex of RBD containing Q498 or H498 binding with mACE2 (**Figures 6C, D**). ELISA results indicated that the RBD of BMA8 and C57MA14 variants has similar binding kinetics to that of the parental SARS-CoV-2 (**Figure 6E**), but the binding affinity was significantly increased between RBD containing H498 mutation and mACE2 receptor in comparison to that between RBD containing Q498 of parental SARS-CoV-2 and hACE2 receptor (**Figure 6F**). In addition, all mACE2 receptor knockout mice (BALB/c^-ACE2^ and C57BL/6J^-ACE2^) survived the infection of SARS-CoV-2 BMA8 and C57MA14 variants, while the normal mice succumbed to the infection (**Figure 6G**). Significant weight loss was observed in BALB/c and C57BL/6J mice, but not in mACE2 receptor knockout mice (**Figure 6H**). In sum, these data indicated that the increased virulence of BMA8 and C57MA14 in mice was likely related to the emergence of Q498H substitution in the RBD of SARS-CoV-2 BMA8 and C57MA14 variants and enhanced binding affinity between RBD containing Q498H mutation and mACE2 receptor.

### Evaluation of the Protective Efficacy of a Bacterium-Like Particle Vaccine Displaying the SARS-CoV-2 RBD in Mice

To value the utility of the two lethal mouse-adapted SARS-CoV-2 variants in mice, we evaluated the protective efficacy of a bacterium-like particle (BLP) vaccine expressing the SARS-CoV-2 RBD in SARS-CoV-2 BMA8-infected aged BALB/c mouse model and C57MA14-infected aged C57BL/6N mouse model. The COVID-19 BLP vaccine was produced as previously described (45). Briefly, SARS-CoV-2 RBD fused with a protein anchor (PA3) was expressed in a baculovirus expression system (**Figure S7A**). The prepared GEM particles were used as vectors to construct COVID-19 BLP by externally displaying the RBD *via* the PA3 (**Figure S7B**). As expected, the surface of the GEM particles has some floc like cotton wool, which is SARS-CoV-2 RBD-PA3 fusion protein, detected by transmission electron microscopy (**Figure S7C**). SARS-CoV-2 RBD bound to GEM particles released strong green fluorescence by immunofluorescence microscopy (**Figure S7D**); sodium dodecyl sulfate polyacrylamide gel electrophoresis (SDS-PAGE) (**Figure S7E**) and a thin layer chromatography scanner (**Figure S7F**) analysis showed that SARS-CoV-2 RBD-PA3 fusion protein is bound to GEM particles with high purity.

**Figure 7:**
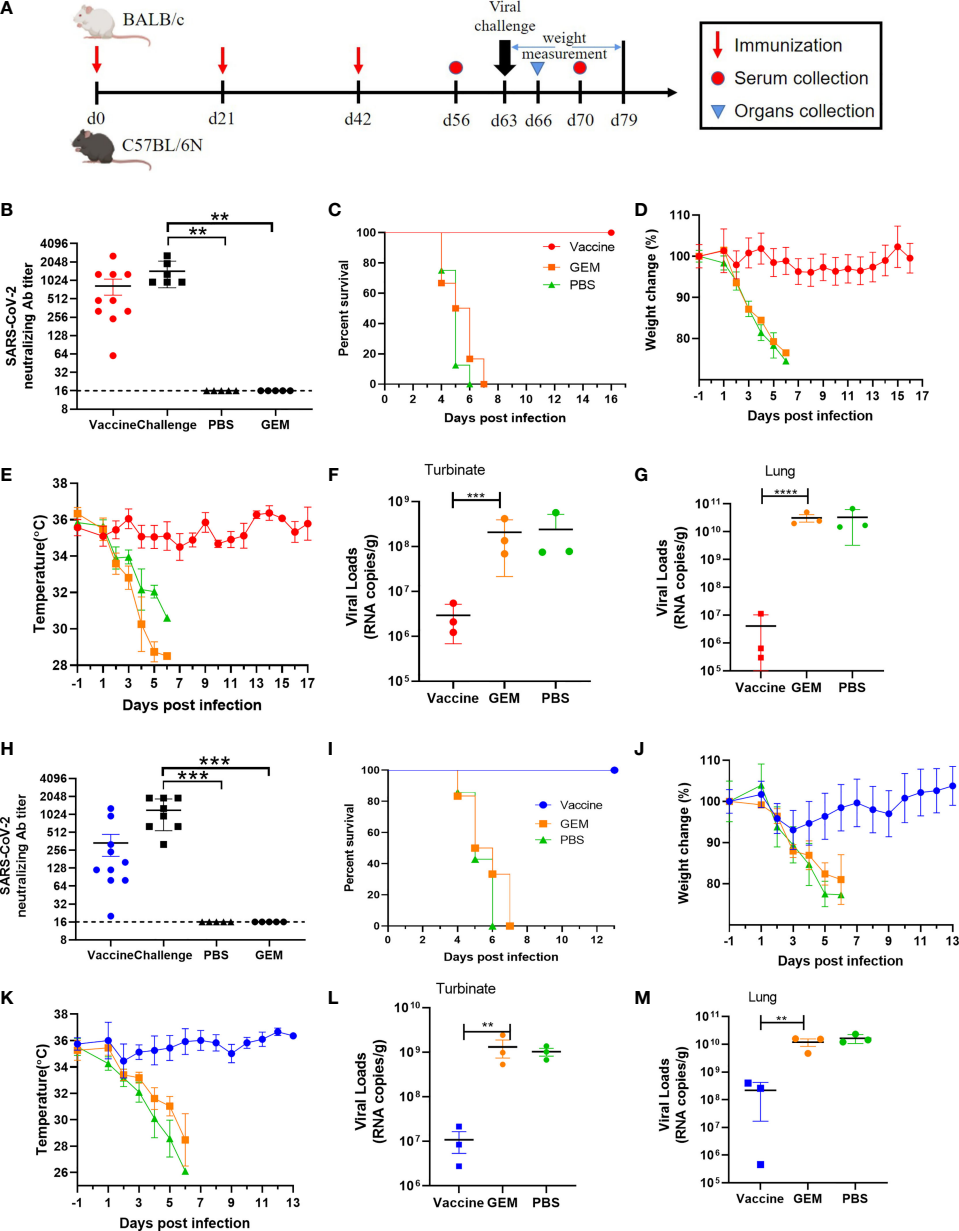
Protection efficacy of the severe acute respiratory syndrome 2 (SARS-CoV-2) bacterium-like particle (BLP) vaccine candidate against BMA8 and C57MA14 infection in aged mice. The vaccination schedule, viral challenge, and characterization of immunologic responses in aged BALB/c and C57BL/6N mice **(A)**. Groups of 9-month-old female BALB/c (n = 10) or C57BL/6N mice (n = 10) were immunized with three doses of 10 µg/dose BLP combined with Freund’s incomplete Freund’s adjuvant (IFA) in 3-week intervals. Empty Gram-positive enhancer matrix (GEM) vector or PBS with same adjuvant was given as negative controls. Serum was harvested at 2 weeks after the last immunization and at 1 week after SARS-CoV-2 challenge to detect the viral neutralizing antibody **(B, H)**. Mice were challenged with 50 LD50 of SARS-CoV-2 BMA8 or C57MA14 *via* i.n. route at 21 days after the third immunization. The survival rate **(C, I)**, weight change **(D, J)**, and body temperature **(E, K)** in each group were monitored daily after BMA8 or C57MA14 challenge. At 3 dpi, three mice in each group were euthanized, and turbinates **(F, L)** and lungs **(G, M)** were sampled for viral RNA loads by qRT-PCR. Data are presented as mean ± SEM. **P < 0.01, ***P < 0.001, ****P < 0.0001.

Here, 9-month-old female BALB/c or C57BL/6N mice were intramuscularly immunized with a dose of 10 µg COVID-19 BLP combined with Freund’s complete adjuvant, followed by two boosters with Freund’s incomplete adjuvant at 3 weeks’ interval. Mice were immunized with GEM particles combined with the same adjuvant while PBS and empty GEM vector were given as control. At 3 weeks after the second booster, aged BALB/c or C57BL/6N mice were intranasally challenged with 50 LD_50_ BMA8 or C57MA14 and were monitored daily for mortality, weight loss, and temperature. The lungs and turbinates were harvested for virological analysis at 3 dpi (**Figure 7A**). Serum samples were harvested 14 days after the third immunization to detect SARS-CoV-2 neutralizing antibodies. As expected, high levels of SARS-CoV-2 neutralizing antibodies were detected in all the COVID-19 BLP-immunized mice (**Figures 7B, H**), and the COVID-19 BLP-immunized mice were 100% protected from two lethal SARS-CoV-2 variant challenges (**Figures 7C, I**). The weight and body temperature did not show any abnormality in aged BALB/c (**Figures 7D, E**), except for the transient and slight weight loss in aged C57BL/6N mice at 2~4 dpi, although the temperature was normal (**Figures 7J, K**). The viral RNA loads in lungs and turbinates from all the immunized mice were significantly reduced compared to the two control groups at 3 dpi (**Figures 7F, G, L, M**). Thus, the above data suggest that our two lethal mouse-adapted variants (BMA8 and C57MA14) could be useful tools to test the immunogenicity of COVID-19 vaccine candidates in mouse models.

## Discussion

Currently, several research groups have enhanced the replication ability of SARS-CoV-2 in mice *via* transgenic technology or continuous passaging *in vivo*. However, most mouse models could not truly reproduce the clinical symptoms of COVID-19 including asymptomatic and mild and severe clinical symptoms presented by COVID-19 patients. Herein, we report two mouse-adapted SARS-CoV-2 variants derived from aged BALB/c mice (BMA8) and aged C57BL/6N mice (C57MA14) *via* serial passaging *in vivo*. More importantly, these two strains perfectly mimicked the clinical symptoms of asymptomatic and moderate to severe COVID-19 patients in genetics-dependent and age/sex-related mice, making up for the deficiency of the existing mouse models of COVID-19.

In our study, the BMA8 variant was used to infect young BALB/c mice and young C57BL/6N mice to recapitulate the asymptomatic COVID-19 patients. No significant symptoms were observed in those BMA8-infected mice, but high viral loads in lungs and turbinates on the early stage of infection were observed. In all mice, the body weight recovered with the decrease in virus load, and virus was undetectable in lungs and turbinates at 14 dpi. The BMA8 variant-infected young BALB/c or C57BL/6N mouse models resembled the symptoms of COVID-19 patients whose SARS-CoV-2 nucleic acid was detectable but without any clinical symptoms. Aged C57BL/6N mice infected with BMA8 mimicked the manifestations of moderate COVID-19 patients, including significant weight loss, decreased temperature, fast breathing, and high viral loads in early infection (10). After BMA8 and C57BL/6N infection, aged BALB/c or C57BL/6N mice displayed severe pneumonia accompanied by fibroplasia, congestion, and edema with hyaline membrane formation, which were similar pathological findings observed in COVID-19 patients (46). The complete blood cell count results in severe COVID-19 were entirely consistent with human patients, while the neutrophilia and lymphopenia accompanied by high-level pro-inflammatory cytokine IL6 were observed in the BMA8- or C57MA14-infected aged BALB/c mice (12, 47, 48). Of note, high viral RNA copies were also detectable in internal organs, including spleen, liver, kidney, heart, brain, and small intestine; however, the tissue tropism was not reflected in SARS-CoV-2 MA10-infected mice (20). This phenomenon may explain that enhanced pathogenic SARS-CoV-2 BMA8 is due to viral replication exacerbating multiple organ damage or failure. The decreased lymphocyte and significantly increased neutrophil remain to be determined in other severe cases of C57MA14 infection. In comparison to MA10 published already, those variants were derived from different hosts. BMA8 was isolated from old BALB/c mice (9-month-old) with 8 passages, while C57MA14 was obtained from old C57BL/6N mice (9-month-old) with 14 passages, but MA10 was developed from young adult BALB/c mice (10-week-old) with 10 passages. Mortality rate of 20%–60% was recorded in young adult BALB/c mice, and nearly 100% mortality rate was shown in aged BALB/c mice (1-year-old), but ameliorated disease and no mortality in C57L/6J mice after SARS-CoV-2 MA10. The pathogenicity of MA10 seemed to be more virulent than BMA8 but weaker than C57MA14. Overall, BMA8 and C57MA14 can reflect the clinical characteristics of asymptomatic, mild, and severe disease of COVID-19 in mice.

Age-related distribution of COVID-19 was reflected in BMA8 and C57MA14-infected mice, with more severe symptoms displayed in aged mice compared with young mice. Although C57MA14 caused lethal disease in mice of all ages, the old mice died earlier than the young ones. Previous studies have provided evidence that host genetic and age variation play protective or pathogenic roles in SARS-CoV disease severity (20, 49). This phenomenon was common in clinical practice. Older people are disproportionately affected by the COVID-19 pandemic because of that age-related decline and dysregulation of immune function, i.e., immunosenescence and inflammation play a major role in contributing to vulnerability to severe COVID-19 outcomes (50). Most advanced-age people accompanied by underlying conditions (e.g., diabetes, hypertension, obesity, and cardiovascular disease) have a higher risk of severe disease and higher mortality of COVID-19. It needs to be determined if aged mice have underlying diseases contributing to the high mortality and morbidity of COVID-19 in the future study.

Serial passaging of virus in mice resulting in adaptive mutations that increase virulence has been well documented in SARS-CoV and MERS-CoV (33, 49, 51). In our study, multiple gene substitutions were confirmed in BMA8 and C57MA14, possibly contributing to gradual viral adaptation and increased infectivity during serial viral passages in mice. RBD in S protein mediates viral entry into the host cell by interacting with hACE2. Mutations in RBD increased the sensitivity between RBD and mACE2 as SARS-CoV-2 does not use mACE2 as its entry receptor (4). In view of all published mouse-adapted SARS-CoV-2 strains, single mutations at Q498H or R493K and two engineered Q498Y and P499T in RBD could promote viral replication in the lungs of mice (41–43). Intriguingly, three mutations including Q493K, Q498Y, and P499T simultaneously occurred in the spike of MA10, while only Q498H mutation was identified in BMA8 and C57MA14. Furthermore, A22D and A36V mutations in E protein were firstly reported in our study but not in MA10. Undoubtedly, amino acid 498 is a key position to enhance the interactions with the mACE2 receptor and increase the pathogenicity. In addition, the mutations in non-structural protein ORF1 including T819I, L1790F, and I65S were speculated to be necessary for viral adaptation in mice because they were concurrently identified in BMA8 and C57MA14. Of note, T67A (A12844G) in nsp9 of ORF1 may play a key role in increasing pathogenicity of BMA8, which leads to a higher fatality rate following its proportion gradually increasing from P1 to P4, while another three mutations have reached the highest ratio before P4. But for C57MA14, P252L (C10809T) mutation in nsp5 of ORF1 is the only one that differed from BMA8 virus, although both variants have four mutations in ORF1. P252L was a non-negligible factor to contribute to the increasing virulence. Gradually increasing mortality from P0 to P14 can be observed as the increasing ratio of P252L, but other mutations have reached the biggest ration before P10, but lethality was not achieved 100%. A22D and A36V substitutions in E took part in multiple mutation pathways to enhance virulence in mice. E protein implicated in the viral pathogenesis and its hydrophobic transmembrane domain are essential for CoV assembly and budding (52, 53). Mutations A22D and A36V in E may provide a reasonable explanation that the C57MA14 variant has higher virulence in C57MA14-infected mice compared to BMA8-infected mice. The C57MA14 causes lethal disease in young mice that was first reported in our study, which is beneficial for the study of the pathogenesis of young COVID-19 patients. Normally, C57MA14 only has A22D substitution in E, but BMA8 combines A22D and A36V substitutes in its genome. The A36V mutation may be a key factor leading to different pathogenicities of the two mutants in different mouse strains. A reverse genetics system will be utilized to investigate how the A36V mutation influences the pathogenicity of those two variants in a further study. A comprehensive model of E protein function will provide a better understanding of SARS-CoV-2 pathogenicity-related factors and may provide a target for therapeutic intervention during SARS-CoV-2 infection. The reverse genetics system of SARS-CoV-2 should be utilized in detail to illustrate how each mutation influences the viral adaptation and pathogenicity in a future study.

Notably, those lethal variants were derived from different hosts. BMA8 was isolated from old BALB/c mice (9-month-old) with 8 passages, while C57MA14 was obtained from old C57BL/6N mice (9-month-old) with 14 passages, but MA10 was developed from young adult BALB/c mice (10-week-old) with 10 passages. Our two-time challenge strategy that the mice were rechallenged 12 h post-infection could explain why we obtained the lethal strain of SARS-CoV-2 passaged for 8 generations and that for 36 generations in another group in aged BALB/c mice (54). Furthermore, lethal strain of C57MA14 was obtained in passage 14 in aged C57BL/6N mice, and the adapted mouse strain was obtained at passage 14 in young BALB/c mice (43). A study has illustrated that mice that received repeated low-dose influenza virus infections showed greater inflammatory responses, more severe lung pathology, and higher viral loads than a single high-dose challenge (55). Repeated virus infections artificially increase selection pressure, promoting SARS-CoV-2 to adapt to the targeted organ of the new host more effectively. In conclusion, repeated virus challenge strategy may quickly drive the coronavirus to evolve in the direction of much more enhanced pathogenicity.

In addition to the age-dependent pathogenicity of SARS-CoV-2, in our profile of immune escape from the SARS-CoV-2 BMA8 or C57MA14, both variants resisted convalescent serum as a comparison to wild-type strain. In addition, several studies have confirmed that N439K, S477N, E484K, and N501Y mutation resulted in immune escape by developing resistance to the SARS-CoV-2 neutralizing antibody (56–58). In light of the above, we must be alert, as larger variants of the spike protein could escape vaccine-induced antibodies. Especially, the spike D614G and N501Y substitutions that are currently prevalent in global SARS-CoV-2 strains exhibit efficient replication in human-to-human transmission, but its effects on viral pathogenesis and transmissibility remain unclear (59–62). Based on these findings, our mouse model could offer a useful tool on the research of transmission and pathogenicity of new SARS-CoV-2 variants.

SARS-CoV-2 BMA8 and C57MA14 models provide a valuable platform for evaluating vaccine candidates. Our results showed that the BLP vaccine elicited a robust neutralization antibody response against BMA8 and C57MA14. Importantly, BLP expressing S RBD of ancestral SARS-CoV-2 could provide 100% protection to the aged BALB/c and C57BL/6N mice against mouse-adapted virus challenge despite Q498H mutation identified in the S RBD, effectively reducing viral load in the upper and lower respiratory tracts of BALB/c and C57BL/6N mice. It was reported that vaccine efficacy will be diminished because aging immune systems can leave the body prone to infection and weaken the response to vaccines (63). BLP vaccine platform provides a new idea for vaccine development. Future studies will be performed to illustrate if the BLP vaccine can induce young mice to produce a similar or higher level of neutralizing antibody compared to that of the aged mice.

Overall, our study has mapped molecular determinants of mouse-adapted SARS-CoV-2 BMA8 and C57MA14 that recapture human COVID-19 phenotypes and age- or host-related disease outcomes, including asymptomatic carrier and moderate and severe patients. The pathogenesis of our two mouse-adapted variants is exquisitely sensitive to host that is under the condition of immunosenescence, which is a common phenomenon in the elderly and recapitulate the disease burden seen in humans. SARS-CoV-2 BMA8 and C57MA14 viruses will promote the use of the mouse models to explore the mechanism of mutation-enhanced transmissibility, host genetics and/or age-dependent high morbidity, and underlying disease-associated high mortality, such as diabetes, obesity, hypertension, and cardiovascular and cerebrovascular disease. Even more importantly, the mouse-adapted infection model in our study can be widely used as a convenient and economical tool in the assessment of vaccines and therapeutic countermeasures.

## Materials and Methods

### Ethics and Biosafety

All procedures involving cells and animals were conducted in biosafety level 3 laboratory (BSL-3) and approved by the animals experimental committee of Laboratory Animal Center, Changchun Veterinary Research Institute (approval number: IACUC of AMMS-11-2020-020). Animals were acclimatized for 3 days prior to infection, given food and water *ad libitum*, and monitored twice daily. Environmental enrichment was also provided in the cages during the study.

### Mouse Strains, Virus, and Cells

Specific pathogen-free 12-week-old or 9-month-old female BALB/c mice, 12-week-old or 9-month-old female C57BL/6N mice, and 9-month-old female C57BL/6J mice were purchased from Beijing Vital River Laboratory (Beijing, China). Nine-month-old mACE2 knockout BALB/c mice (BALB/c^-ACE2^) were purchased from Cyagen Biosciences (Guangzhou, China). Nine-month-old mACE2 knockout C57BL/6J mice (C57BL/6J^-ACE2^) were purchased from Shanghai Model Organisms Center (Shanghai, China). All animals were maintained in the Animal Care Facilities of Changchun Veterinary Research Institute. The SARS-CoV-2 Wuhan01 was originally isolated from a COVID-19 patient in Wuhan (BetaCov/Wuhan/AMMS01/2020) and passaged on Vero E6 cells. Vero E6 cells were cultured in Dulbecco’s modified Eagle’s medium (DMEM; GIBCO, Grand Island, NY) supplemented with 10% fetal bovine serum (FBS; Biological Industries, Israel) and 1% penicillin-streptomycin (Sigma-Aldrich).

### *In Vivo* Infection

Three 9-month-old female BALB/c or C57BL/6N mice were lightly anesthetized with isoflurane and intranasally (i.n.) inoculated with 10^5^ median tissue culture infectious dose (TCID_50_) of SARS-CoV-2 Wuhan01 in a volume of 50 µl. Subsequently, aged mice were rechallenged with the same dose 12 h after infection. Three days after infection, the mice were anesthetized and lungs were harvested and homogenized with 400 µl DMEM containing 10% FBS and 1% penicillin-streptomycin. The lung homogenate was clarified by high-speed centrifugation at 12,000 g for 15 min, and the supernatant was administered intranasally to another three aged mice, and the lung homogenates were used as inoculation with blind viral titers for continued serial passage by intranasally inoculating in a volume of 50 µl per mouse and reinoculated with the same volume at 12 h post-infection. The process of intranasal inoculation was repeated 8 times in aged BALB/c mice and 14 times in aged C57BL/6N mice.

In subsequent *in vivo* infection, all animals were intranasally infected with a predetermined dose of SARS-CoV-2 and monitored daily for signs of disease, which included weight loss, temperature, mortality, physical activities, and food/water intake. Some mice were euthanized, and the target samples were harvested according to the predetermined time.

### Extraction of Viral RNA and Quantitative RT-PCR

Tissue homogenates were prepared by homogenizing whole tissue with an electric homogenizer for 300 s in 500 µl DMEM. The supernatant was collected after the homogenates were centrifuged at 12,000 rpm for 10 min at 4°C. Viral RNA was extracted using the QIAamp^®^ viral RNA Mini Kit according to the manufacturer’s protocol. Viral RNA quantification was detected by quantitative reverse transcription PCR (RT-qPCR) targeting the N gene of SARS-CoV-2. RT-qPCR was performed with Premix Ex Taq (Takara, China) with the following primers and probes: NF (5′-GGGGAACTTCTCCTGCTAGAAT-3′); NR (5′-CAGACATTTTGCTCTCAAGCTG-3′); and NP (5′-FAM-TTGCTGCTGCTTGACAGATT-TAMRA-3′).

### Quantification of Viral Loads by TCID_50_

Lung homogenate supernatant was serially diluted in DMEM and added into Vero E6 cells in 96-well plates. The plates were incubated for 1 h at 37°C with 5% CO_2_; the inoculation was replaced with DMEM containing 2% FBS and 1% penicillin-streptomycin. After incubating for 72 h, the TCID_50_ was detected by the cytopathic effect (CPE).

### Whole-Genome Sequencing of Mouse-Adapted SARS-CoV-2

Reverse transcription of viral RNA (extracted as described above) was carried out using the SuperScript III RT enzyme (Thermo Fisher Scientific, Waltham, MA, USA) and random hexamer primers (Thermo Fisher Scientific, Waltham, MA, USA) following the manufacturer’s instructions. Subsequent PCR reactions were carried out using the Phusion Green Hi-Fidelity PCR mix (Thermo Fisher Scientific, Waltham, MA, USA) under the following conditions: 35 cycles of 98°C for 30 s, 65°C for 30 s, and 72°C for 90 s with a final extension at 72°C for 10 min. Amplicons were purified by gel electrophoresis and quantified using the PicoGreen dye, and fragments from the same passage were pooled at equimolar concentration using a BioMek FX. Library construction was done using the Nextera DNA Sample Preparation kit (24-Sample) (Illumina, San Diego, CA, USA) as per the manufacturer’s instructions. Sequencing was carried out using a MiSeq sequencer (Illumina, San Diego, CA, USA) using the MiSeq Reagent Kit v3 (600 cycles) (Illumina, San Diego, CA, USA). Paired reads from each passage were aligned to a reference sequence (GenBank accession no. NC_045512.2) using Bowtie 2 version 2.0.5. Conversion to bam files, sorting, and indexing were performed using Samtools version 0.1.18. Variant calling was performed using freebayes version 0.9.8 using a ploidy of 4, a max complex gap of 0, a minimum quality score of 30, a minimum frequency of 5%, minimum coverage of 200-fold, and to ignore insertions and deletions. The frequencies of variants were determined as the number of reads with a specific variant over the depth at that location. Finally, coverage was determined using bedtools version 2.25.0.

Clonal isolates of P8 from aged BALB/c mice and P14 from C57BL/6N mice were plaque purified from a plaque assay of infected mouse lung homogenates on Vero E6 cells, respectively, generating a passage 1 SARS-CoV-2 BMA8 and SARS-CoV-2 C57MA14. Both isolated plaques were propagated and titered on Vero E6 cells for all subsequent studies.

To obtain the single step growth curves of SARS-CoV-2 Wuhan01, BMA8, and C57MA14, Vero E6 cells were infected with a multiplicity of infection (MOI) of 1 for 1 h at 37°C with 5% CO_2_. Cells were washed three times with DMEM, and complete medium containing 2% FBS and 1% penicillin-streptomycin was added. The supernatant was collected at designated times and stored at -80°C for the viral load test.

The sera from 32 individuals infected with SARS-CoV-2 Wuhan1 (BetaCoV/Wuhan/AMMS01/2020) were obtained from the Affiliated Hospital of University of South China (Changsha, China). All donors provided written informed consent for only the research use of the blood. The sera were serially diluted in DMEM and incubated with 100 TCID_50_ of SARS-CoV-2 Wuhan01, BMA8, or C57MA14 separately. After incubation at 37°C for 1 h, aliquots were added to Vero E6 cell (2 × 10^5^ cells/well) in 96-well plates. CPE of the cell was recorded at 3 dpi under microscopes, and the neutralizing antibody titers were calculated by the dilution number of 100% protective condition.

### Complete Blood Cell Counts

To determine the complete blood cell counts, samples were analyzed using an auto hematology analyzer (BC-5000vet, Mindray, China) according to the manufacturer’s instructions.

### Histology and Immunohistochemistry

The organs were fixed in 10% phosphate buffered formalin, and paraffin sections were prepared routinely at 5 µm. The sections were stained with hematoxylin and eosin (H&E) for histopathologic examination. For immunohistochemistry (IHC), paraffin-embedded tissues were quenched for 10 min in aqueous 3% hydrogen peroxide. Mouse monoclonal anti-SARS-CoV-2 N antibody produced in mice was applied to the section as the primary antibody (Sino Biological Inc., China) at a 1:500 dilution for 30 min. Sections were visualized using a horseradish peroxidase (HRP)-labeled polymer, Envision^®^+ system (anti-mouse) (Dako, USA), subjected to reaction with the chromogen diaminobenzidine (DAB) and counterstained with Gill’s hematoxylin.

For multiplex immunofluorescent assay, primary antibody was incubated overnight at 4°C. Afterward, sections were incubated with the HRP-conjugated secondary antibody for 20 min at room temperature. After washing, polymer tagged HRP mediated the covalent binding of a different fluorophore sequentially and coupled with tyramide signal amplification (TSA) for detection. Subsequently, the primary and secondary antibodies were thoroughly eliminated by heating for 10 s at 95°C using microwave. In a serial fashion, each antigen was labeled by distinct fluorophores. At last, sections were counterstained with 4',6-diamidino-2-phenylindole (DAPI). Multiplex antibody panels applied in this study include ACE2 (Abcam, England, 1:200) and SARS-CoV-2 S (Sino Biological, China, 1:2,000). After all the antibodies were detected sequentially, the slides were imaged using the confocal laser scanning microscopy platform LEICA SP8.

### Cytokine Analysis

Groups of 10 9-month-old female BALB/c mice or C57BL/6N mice were intranasally challenged with 50 LD_50_ BMA8 or C57MA14 separately. Groups of 7 9-month-old female BALB/c mice or C57BL/6N mice were infected with PBS as control. After 3 days, the cytokine profiles in mouse sera were measured using V-PLEX Custom Mouse Cytokine10-plex kits according to the manufacturer’s protocol. Inflammatory cytokines in this panel included IFN-γ, IL-1β, IL-2, IL-4, IL-5, IL-6, IL-10, IL-12p70, KC/GRO, and tumor necrosis factor (TNF)-α. The data were collected by MESO QuickPlex SQ 120 plate reader (MSD) and analyzed with Discovery Workbench Software (v4.0, MSD).

### ELISA

ELISA was performed to detect the binding affinity between RBD and ACE2. Briefly, ELISA plates were precoated with mACE2 or hACE2 (1 μg/ml) overnight at 4°C and blocked with 2% fat-free milk in phosphate buffered solution (PBST) for 2 h at 37°C. Serially diluted His-labeled RBD with Q498 and RBD with H498 that have the same initial concentration were added to the plates and incubated for 2 h at 37°C. After four washes, the bound RBDs were detected by incubation with HRP-conjugated mouse anti-His IgG antibody (Abcam, USA, 1:5,000) for 1 h at 37°C. The reaction was visualized by addition of substrate 3,3’,5,5’-tetramethylbenzidine (TMB) (Sigma, USA) and stopped by adding H_2_SO_4_. The absorbance at 450 nm was measured by an ELISA plate reader (Tecan, USA).

### Vaccine Studies

The SARS-CoV-2 BLPs were produced as previously described (45). The recombinant RBD-linker-PA3 protein was expressed with the Bac-to-Bac baculovirus expression system. The code-optimized recombinant DNA encoding SARS-CoV-2 spike protein residues 319-537 fused with PA3 containing three LysMs (*L. lactis* MG1363) was inserted into the baculovirus transfer vector pFastBac1-HBM plasmid (Invitrogen, Carlsbad, CA, USA). The GEM particles externally display the SARS-CoV-2 RBD through the PA3. The binding GEM particles were detected by standard error of the mean (SEM) and incomplete Freund’s adjuvant (IFA). The purity of the recombinant protein displayed on the surface of GEM particles was examined by SDS-PAGE and thin-layer chromatography scanning.

Nine-month-old female BALB/c mice and C57BL/6N mice were immunized with SARS-CoV-2 BLPs. Briefly, BALB/c mice and C57BL/6N mice were randomly separated into four groups and vaccinated with 10 µg SARS-CoV-2 BLPs in 50 μl PBS mixed with IFA *via* intramuscular way. Mice in the control group were given 50 μl PBS mixed with 50 μl IFA. All groups received a second and third booster immunization at 3 and 6 weeks following the primary immunization. Sera were collected 2 weeks after the last booster immunization and 1 week after the SARS-CoV-2 challenge to detect viral neutralizing antibodies as described below. Three weeks after the second booster, BALB/c mice or C57BL/6N mice were intranasally challenged with 50 LD_50_ SARS-CoV-2 BMA8 or C57MA14 separately. The body weight of all mice was observed daily until 16 days. At 3 dpi, three mice in each group were sacrificed, and lungs were collected to analyze the viral RNA loads.

### SARS-CoV-2 Neutralization Assay

Neutralizing titers of mice sera were carried out by using SARS-CoV-2 Wuhan01 and two variants (BMA8 and C57MA14) as previously described (64). Briefly, mouse sera were serially diluted in DMEM and incubated with an equal volume of SARS-CoV-2 containing 100 TCID_50_. After incubation at 37°C for 1 h, aliquots were added to 50 μl of 1 × 10^4^ Vero E6 cells in 96-well plates. The cells were observed daily for the presence or absence of virus-caused CPE and recorded at 3 dpi under microscopes, and the neutralizing antibody titers were calculated as the highest dilution of sera that completely inhibits virus-caused CPE. Serum neutralizing antibody titer was defined as the reciprocal of the highest dilution showing a 100% CPE reduction compared to the virus control. Virus-only controls and cell-only controls were included in each plate.

### Data Analysis

All statistical analyses were made using GraphPad Prism 8.0 (GraphPad Software, La Jolla, CA, USA). All data are shown as mean ± standard error of the mean (SEM). Statistical significance was calculated using one-way ANOVA. n.s. indicates not significant, and p < 0.05 was considered statistical significance.

## Data Availability Statement

The data presented in the study are deposited in the Genbank repository, accession number OL913103 and OL910104.

## Ethics Statement

All procedures involving cells and animals were conducted in biosafety level 3 laboratory (BSL-3) and approved by the animals experimental committee of Laboratory Animal Center, Changchun Veterinary Research Institute (approval number: IACUC of AMMS-11-2020-020).

## Author Contributions

YG conceived the study and designed the experiments. FY and EL performed all animal experiments in Level 3 laboratory. FY, EL, WW, and HP interpreted and imaged the data. FY, EL, TW, YL, JL, and NF performed *in vitro* experiments and analyzed the data. FY and EL wrote the article. YG, SY, and XX supervised the project and modified the article. TQ, RS and SW performed RNA extraction and collected the data. YZ modified the article. All authors contributed to the article and approved the submitted version.

## Funding

This work was supported by the National Science and Technology Project (2020YFC0846100).

## Conflict of Interest

The authors declare that the research was conducted in the absence of any commercial or financial relationships that could be construed as a potential conflict of interest.

## Publisher’s Note

All claims expressed in this article are solely those of the authors and do not necessarily represent those of their affiliated organizations, or those of the publisher, the editors and the reviewers. Any product that may be evaluated in this article, or claim that may be made by its manufacturer, is not guaranteed or endorsed by the publisher.
