# Supplementary material for: Characterization of Two Heterogeneous Lethal Mouse-Adapted SARS-CoV-2 Variants Recapitulating Representative Aspects of Human COVID-19
Source: Front Immunol. 2022 Feb 7;13:821664. doi: 10.3389/fimmu.2022.821664 (PMC8858946; doi:10.3389/fimmu.2022.821664)
Supplement: Supplementary file 1 [file DataSheet_1.docx]

**Figure S1**


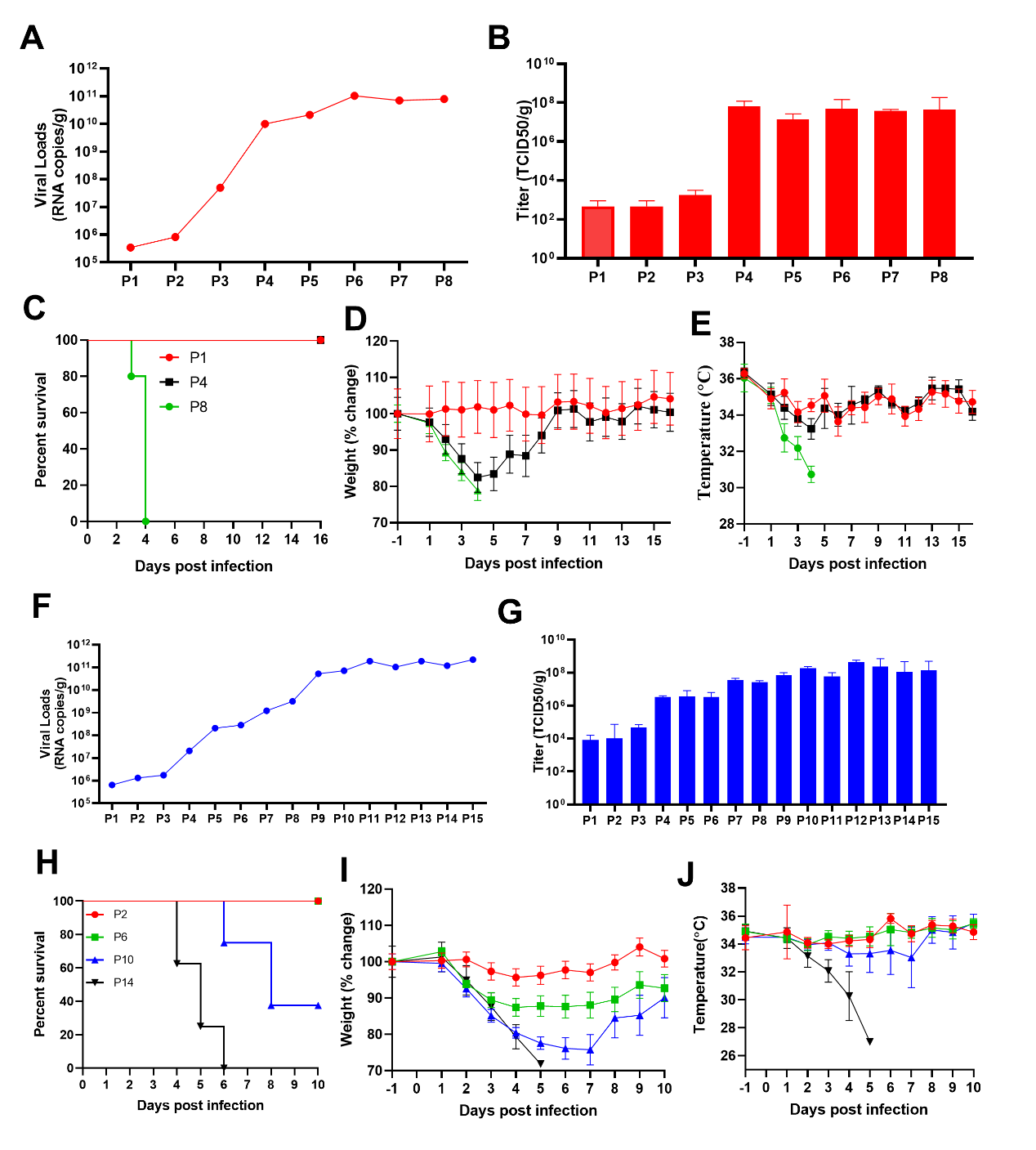


Figure S1, related to figure 1. Development of mouse-adapted strains of SARS-CoV-2 in aged BALB/c and C57BL/6N mice, respectively.

SARS-CoV-2 genomic RNA copies detected by qRT-PCR (A) and lung viral titers detected by TCID_50_ (B) in mouse lung homogenates at 3 dpi throughout serial passage of SARS-CoV-2 in 9-month-old female BALB/c mice infected with 10^5^TCID_50_ SARS-CoV-2 at passage 1 (P1), or blind titer for P2~P8. Data are presented as means ± standard error of the means (SEM) (n=3). The survival rate (C), weight change (D), and body temperature (E) were observed daily after P1, P4 or P8 of SARS-CoV-2 infection in 9-month-old female BALB/c mice. Data are presented as means ± SEM (n=8).

SARS-CoV-2 genomic RNA copies detected by qRT-PCR (F) and lung viral titers detected by TCID_50_ (G) in mouse lung homogenates at 3 dpi throughout serial passage of SARS-CoV-2 Wuhan01 in 9-month-old female C57BL/6N mice infected with 10^5^TCID_50_ SARS-CoV-2 at P1, or blind titer for P2~14. Data are presented as means ±SEM (n=3). The survival rate (H), weight change (I) and body temperature (J) were observed daily after P2, P6, P10 or P14 of SARS-CoV-2 infection in 9-month-old female C57BL/6N mice. Data are presented as means ± SEM (n=8).

**Figure S2**


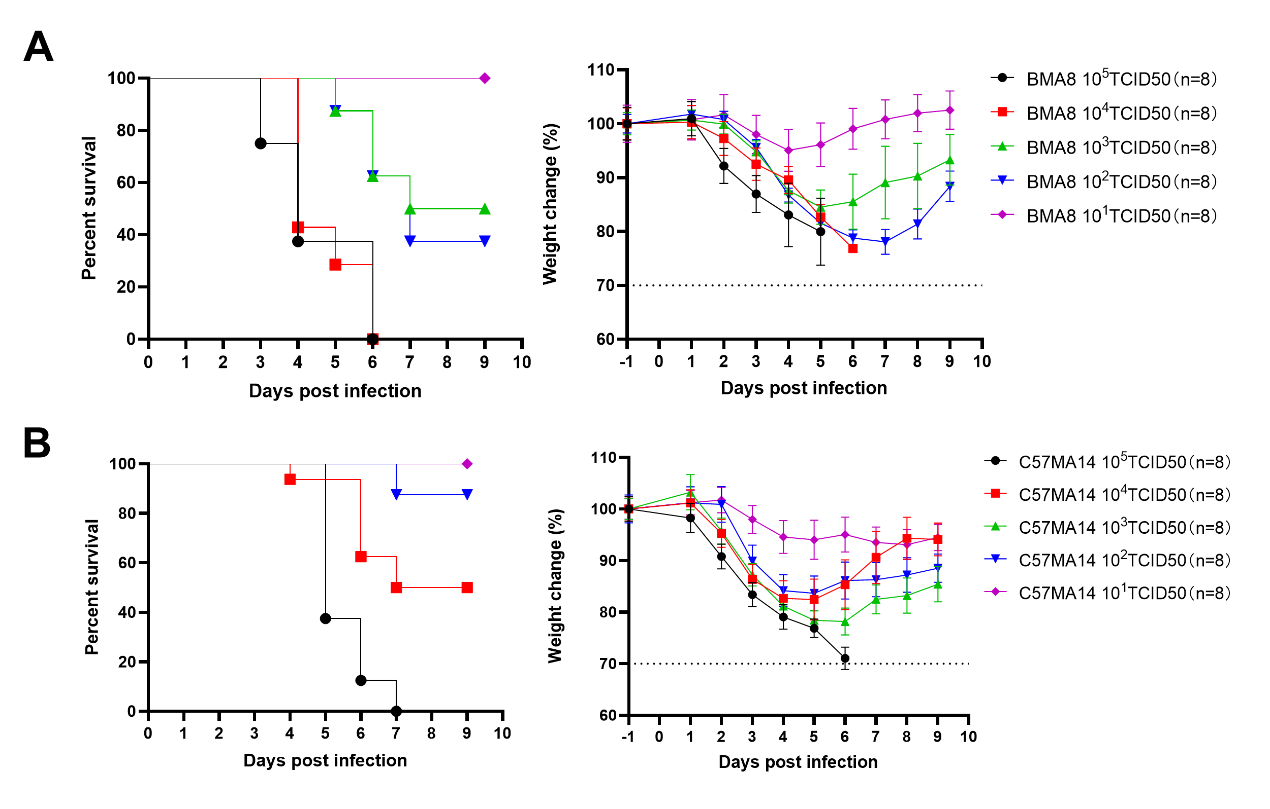


Figure S2, related to figure 2. Dose-dependent pathogenic potential of SARS-CoV-2 BMA8 and C57MA14 in aged BALB/c and C57BL/6N mice.

9-month-old female BALB/c mice (n=8) were infected with 10^1^, 10^2^, 10^3^, 10^4^, 10^5^ TCID_50_ SARS-CoV-2 BMA8. The survival rate and weight change were monitored daily after SARS-CoV-2 infection (A). Meanwhile, aging C57BL/6N mice (n=8) were infected with 10^1^, 10^2^, 10^3^, 10^4^, 10^5^ TCID_50_ SARS-CoV-2 C57MA14, and the survival and weight loss were also monitored daily after SARS-CoV-2 infection (B). Dotted line represents weight loss criteria for human euthanasia. Data are presented as means ± SEM (n=8).

**Figure S3**


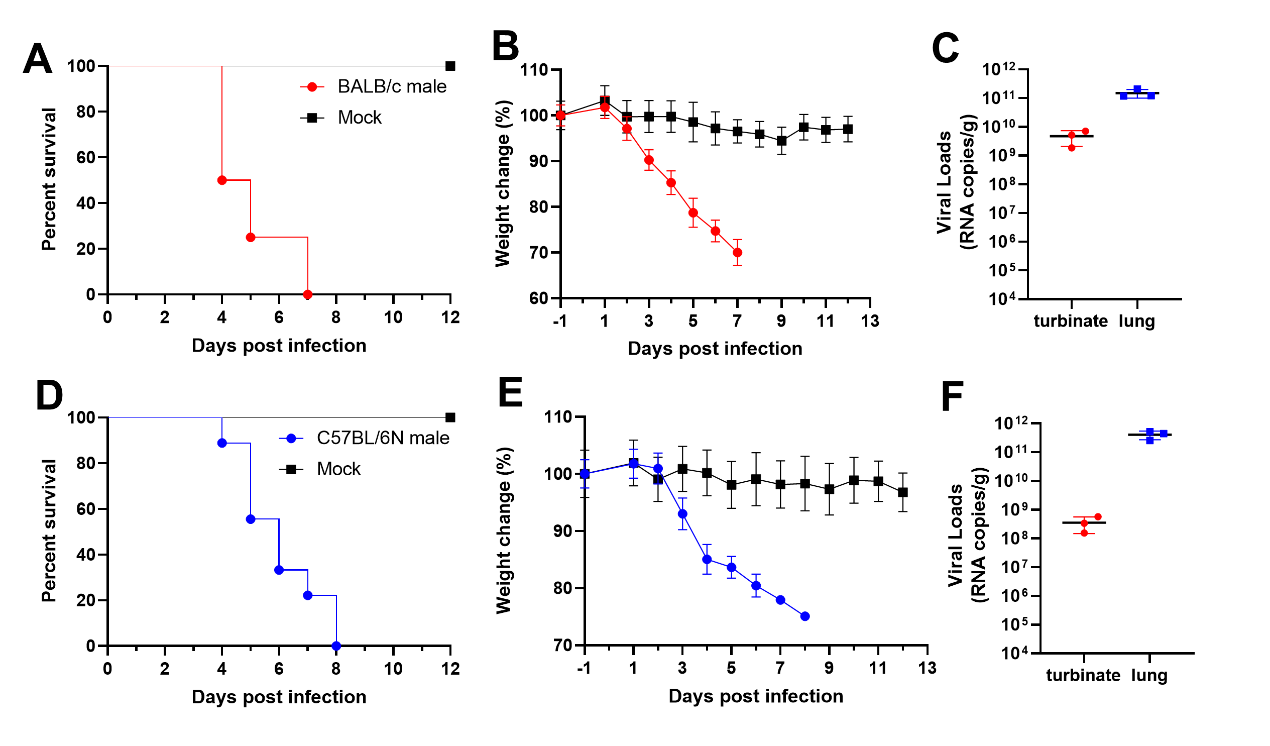


Figure S3，related to figure 2. Evaluation of SARS-CoV-2 BMA8 and C57MA14 in aged male BALB/c mice and C57BL/6N mice.

Groups of ten 9-month-old male BALB/c or C57BL/6N mice were infected with 50 LD50 SARS-CoV-2 BMA8 or 50 LD50 SARS-CoV-2 C57MA14, respectively. Groups of ten 9-month-old male BALB/c mice and male C57BL/6N mice were infected with PBS as control. The deaths (A and D) and weight (B and E) were daily monitored. Three mice in each group were sacrificed to collect turbinates and lungs for viral RNA loads analysis (C and F).

**Figure S4**


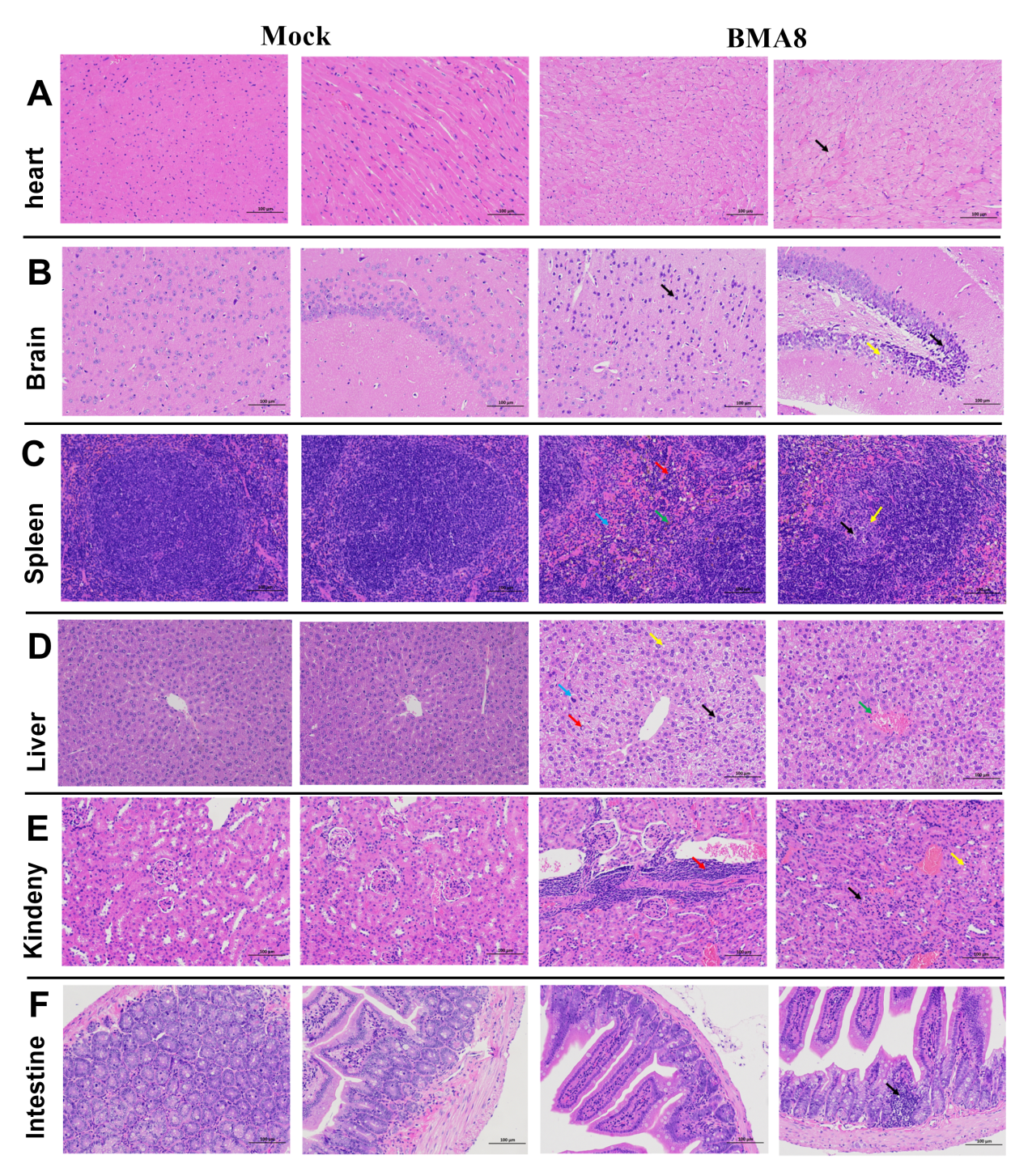


Figure S4, related to figure 3. Histopathology findings in aged BALB/c mice infected with 50 LD_50_ SARS-CoV-2 BMA8.

To investigate the organ pathology excepted lung tissues after viral infection, aging BALB/c mice were infected with 50 LD_50_ SARS-CoV-2 BMA8 or PBS (Mock) and the organs including heart, brain, spleen, liver, kidney and intestine were also collected at 3 dpi. (A) The myocardial cells are swollen, the cytoplasm is loose and light stained in heart tissues after viral infection (black arrow). (B) Brain changes are characterized by neuronal shrinkage, cell staining deepened and the obscure boundary between nucleus and cytoplasm (black arrow) and neuronal cytoplasmic vacuolization (yellow arrow). (C) There are scatter areas of nuclear pyknosis, deep staining or fragmentation (black arrow), expansion of germinal center (yellow arrow), extramedullary hematopoiesis foci in the red pulp (red arrow) with scattered neutrophil infiltration (blue arrow) and brownish yellow granules in the red pulp (green arrow). (D) Ballooning degeneration and swelling, nucleus in the middle, cytoplasmic vacuolation (black arrow), round vacuoles of different sizes in cytoplasm (yellow arrow), venous congestion (green arrow) are usually observed; Intranuclear inclusion body (red arrow) and multinucleated giant cell (blue arrow) are occasionally observed.(E) The atrophy of renal capsule cavity (black arrow) and lymphocytic foci around the blood vessels (red arrow) are usually observed; Vacuolar degeneration of renal tubular epithelial cells and round vacuoles of different sizes in the cytoplasm can be occasionally seen (yellow arrow). (F) lymphocytic infiltration can be seen in local areas (black arrow). Scale bar, 100 µm.

**Figure S5**


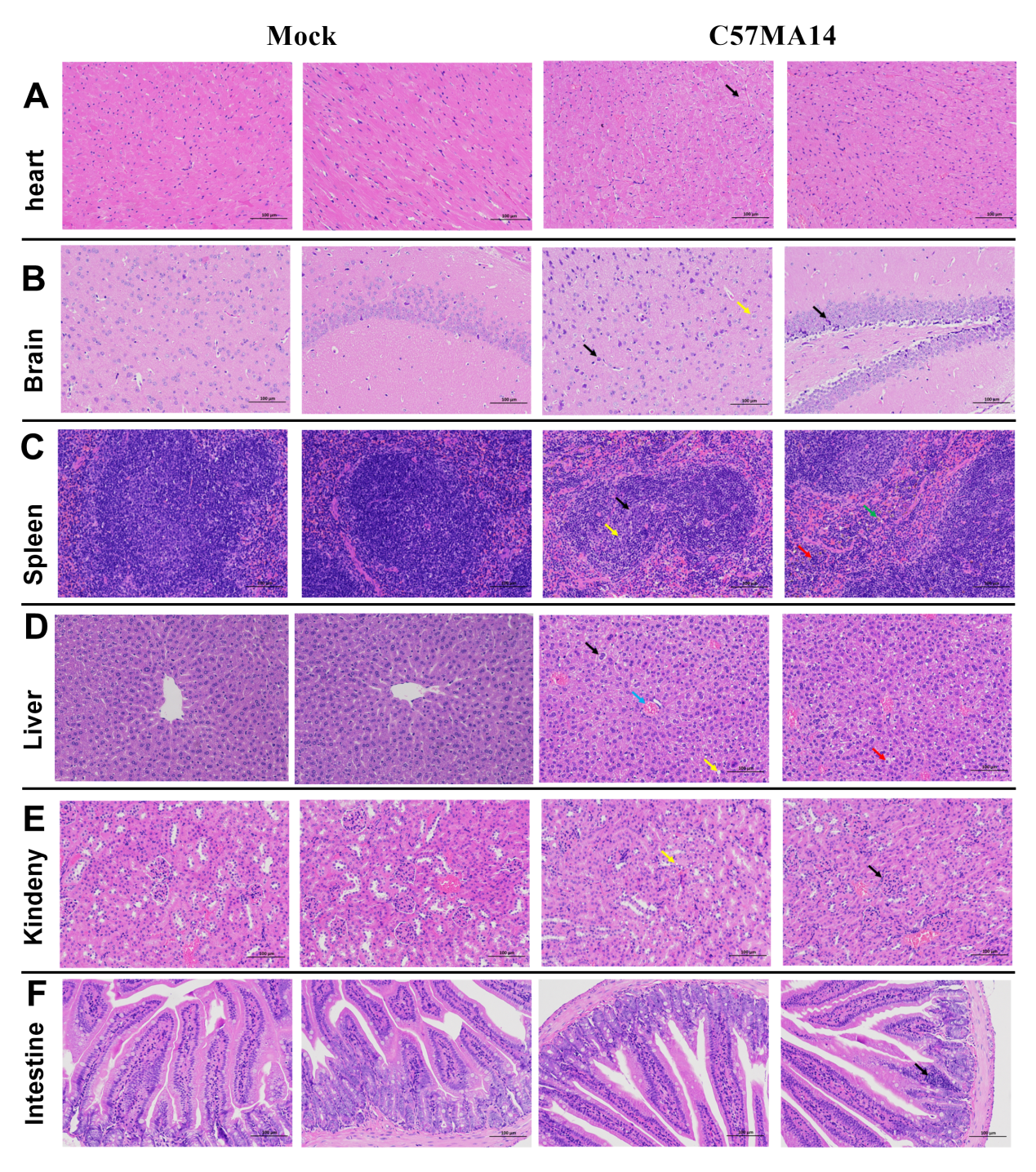


Figure S5, related to figure 3. Histopathology findings in aged C57BL/6N mice infected with 50 LD_50_ SARS-CoV-2 C57MA14.

To investigate the organ pathology excepted lung tissues after viral infection, aging C57BL/6N mice were infected with 50 LD_50_ SARS-CoV-2 C57MA14 or PBS (Mock) and the organs including heart, brain, spleen, liver, kidney and intestine were also collected at 3 dpi. (A) The swollen myocardial cells and loosen and light stained cytoplasm can be occasionally observed in heart tissues (black arrow). (B) The neuronal shrinkage, cell staining deepened and the obscure boundary between nucleus and cytoplasm (black arrow) and neuronal cytoplasmic vacuolization (yellow arrow) are occasionally observed in brain tissues. (C) Spleen changes are characterized by expansion of germinal center (black arrow), nuclear pyknosis or fragmentation (yellow arrow), extramedullary hematopoiesis foci in the red pulp (red arrow), and brownish yellow granules in the red pulp (green arrow). (D) The cytoplasm rarefaction (black arrow) and venous congestion (blue arrow) are usually observed; The ballooning degeneration and swelling, nucleus in the middle, cytoplasmic vacuolation (red arrow) and round vacuoles of different sizes in cytoplasm (yellow arrow) are occasionally observed.(E) The atrophy of renal capsule cavity (black arrow), vacuolar degeneration of renal tubular epithelial cells and round vacuoles of different sizes in the cytoplasm (yellow arrow) can be seen in many areas. (F) lymphocytic infiltration can be seen in local areas (black arrow). Scale bar, 100 µm.

**Figure S6**


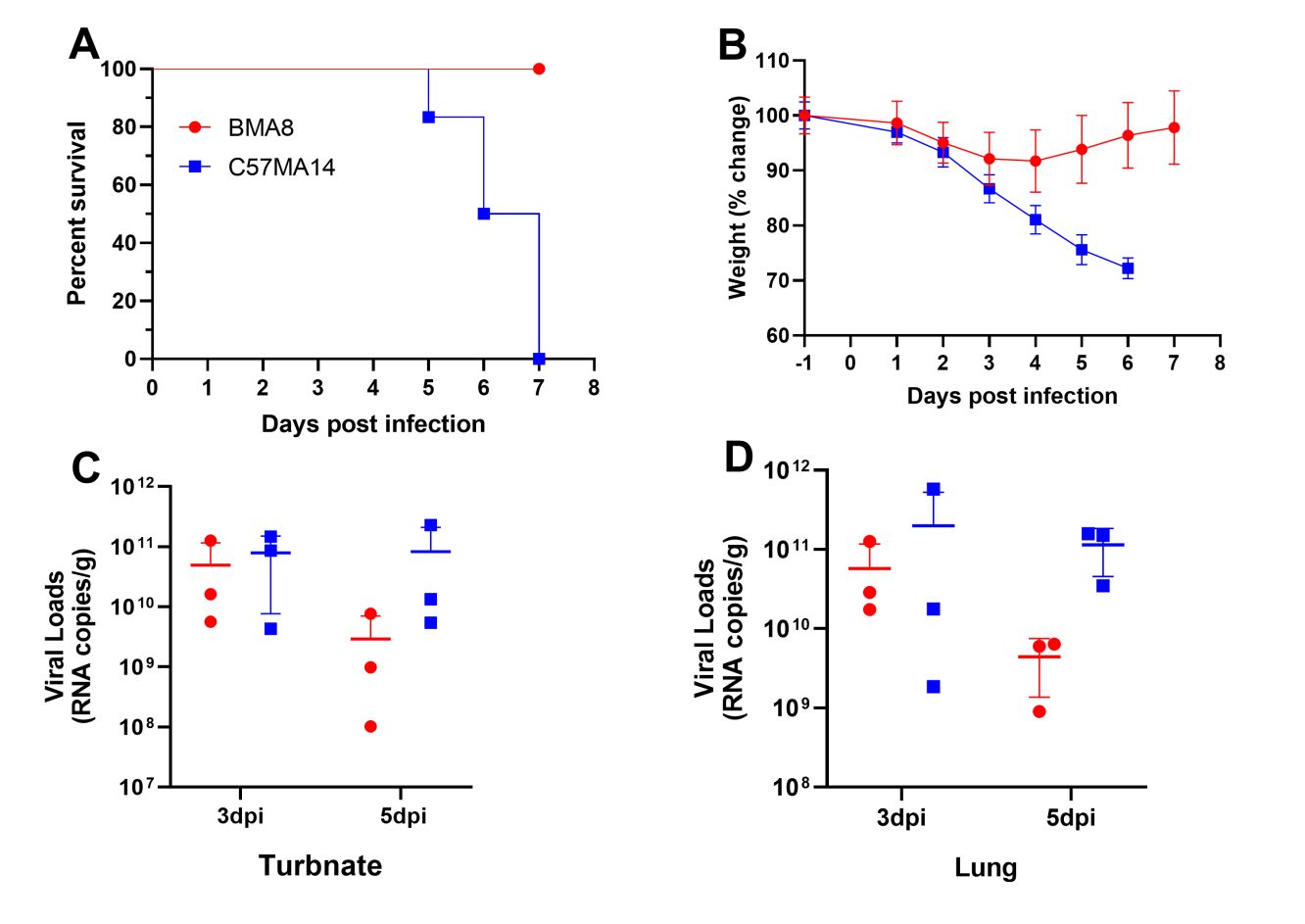


Figure S6, related to figure 5. Evaluation the pathogenicity of SARS-CoV-2 BMA8 and C57MA14 in C57BL/6J mice. Groups of 9-month-old female C57BL/6J mice (n=8) were infected intranasally with 50 LD_50_ of SARS-CoV-2 BMA8 in a volume of 50 ul. The survival rate and weight change in each group (n=5) were monitored daily after infection (A,B). At 3, 5, and 7 dpi, three mice of each group were euthanized, and turbinates and lungs were sampled for virus RNA loads by qRT-PCR (C,D).

**Figure S7**


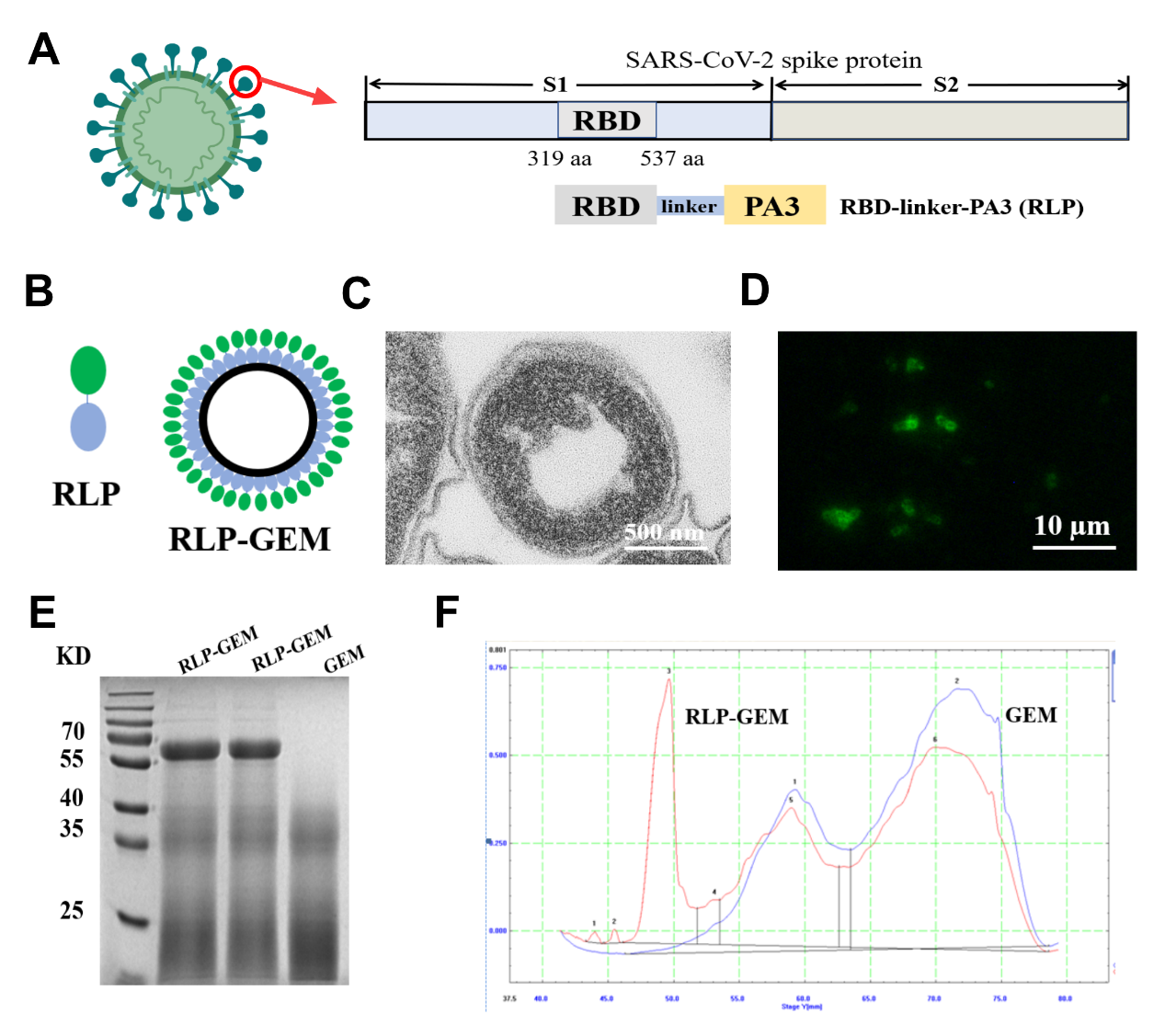


Figure S7, related to figure 7. Construction and characterization of the bacterium-like particle vaccine displaying the RBD of SARS-CoV-2 Wuhan01.

(A) Schematic illustration of the RBD-linker-PA3 fusion protein. (B) Schematic diagram of the RBD-based SARS-CoV-2 bacterium-like particle (BLP) vaccine. (C) The RBD-based SARS-CoV-2 BLP was identified by transmission electron microscope. (D) RBD-linker-PA3 fusion protein displayed on the surface of BLP evaluated by indirect fluorescence assay (IFA) using a mouse anti-SARS-CoV-2 S polyclonal antibody. (E) RLP-GEM and GEM was distinguished by sodium dodecyl sulfate-polyacrylamide gel electrophoresis (SDS-PAGE). (F) The purification of RLP-GEM was identified by a thin layer chromatography scanner.
